# Sequential carbonyl derivatives and hydrazone adduct formation on myeloperoxidase contribute to development of ANCA vasculitis

**DOI:** 10.1172/JCI178813

**Published:** 2025-02-27

**Authors:** Gang Xi, Elizabeth A. Mclnnis, Olivier Lardinois, Peiqi Hu, John S. Poulton, Meghan E. Free, Dhruti P. Chen, Evan M. Zeitler, Eveline Y. Wu, Nicole M. Orzechowski, Vimal K. Derebail, J. Charles Jennette, Ronald J. Falk

**Affiliations:** 1 University of North Carolina Kidney Center, Division of Nephrology and Hypertension, Department of Medicine, University of North Carolina at Chapel Hill, Chapel Hill, North Carolina, USA.; 2 Mass Spectrometry Research and Support Group, National Institute of Environmental Health Sciences, NIH, Research Triangle Park, North Carolina, USA.; 3 Department of Pathology and Laboratory Medicine,; 4 Division of Pediatric Rheumatology, Department of Pediatrics,; 5 Division of Rheumatology, Allergy, and Immunology, Department of Medicine, University of North Carolina at Chapel Hill, Chapel Hill, North Carolina, USA.

**Keywords:** Autoimmunity, Nephrology, Autoimmune diseases, Vasculitis

## Abstract

Drug-induced autoimmune diseases are increasingly recognized, although mechanistic insight into disease causation is lacking. Hydralazine exposure has been linked to autoimmune diseases, including antineutrophil cytoplasmic autoantibody (ANCA) vasculitis. Our hypothesis posits that hydralazine covalently binds to myeloperoxidase (MPO), triggering the autoimmune response in ANCA vasculitis. In vitro, we observed formation of carbonyl derivatives on amine groups in the presence of acrolein. This facilitated the subsequent binding of hydralazine to heme-containing proteins, including MPO, via a Michael addition. Our studies demonstrated that carbonyl derivatives and hydrazone adducts induced conformational changes in the MPO heavy chain, potentially changing its immunogenicity. We identified hydrazone adducts on circulating MPO in patients with hydralazine-associated ANCA vasculitis. These patients exhibited elevated anti-MPO IgM levels, while anti-MPO IgG levels were comparable between hydralazine-associated and nonhydralazine-associated vasculitis patients. IgM isolated from patients with hydralazine-associated MPO ANCA demonstrated a heightened affinity to hydralazine-modified MPO and activated neutrophil-like HL-60 cells. Hydralazine-modified MPO was pathogenic, as demonstrated by splenocyte transfer in a mouse model of ANCA vasculitis. Our findings unveil a mechanism of drug-induced autoimmunity wherein stepwise chemical modifications of MPO lead to conformational changes and hydrazone adduct formation, producing a neoantigen that generates pathogenic autoantibodies.

## Abstract 2

This study provides a framework for understanding the pathogenesis of hydralazine-associated autoimmune diseases and may illuminate a certain set of mechanisms of autoimmunity in general.

## Graphical abstract

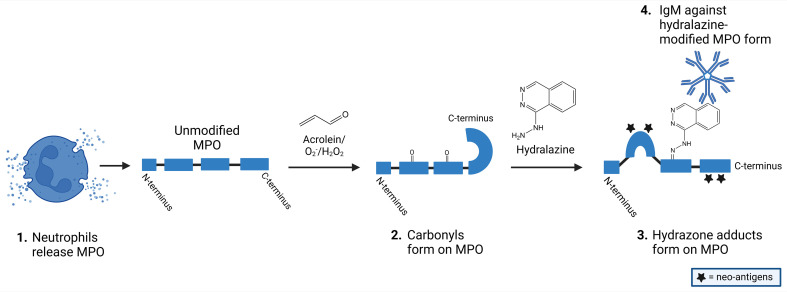

## Introduction

Since the initial report of sulfadiazine-associated lupus-like symptoms in 1945 (1), drug-induced autoimmunity has been a well-recognized phenomenon. Among the many implicated drugs, hydralazine is a common offender. Despite recognition of this phenomenon for decades, the underlying mechanisms that lead to drug-induced autoimmunity remain poorly understood. As drug-induced autoimmunity can be viewed as an “experiment of nature,” understanding the mechanisms of disease induction are key to also understanding other idiopathic autoimmune disease.

The etiology of autoimmune disease has been attributed to many factors that include, but are not limited to, genetic and environmental factors (2–5). An extensive array of potential contributors poses a formidable challenge in unraveling the intricacies leading to specific immune dysfunction and disease manifestation. Drug-induced autoimmunity provides a window into how an endogenous protein may become an autoantigen. Many theories have been proposed to explain this phenomenon (6). In hydralazine-induced antineutrophil cytoplasmic antibody (ANCA) vasculitis, one theory suggests that hydralazine accumulates in neutrophils, binds to myeloperoxidase (MPO), and prompts cytotoxic product generation, cell death, and the exposure of typically sequestered antigen (7). However, these hypotheses lack direct evidential support.

ANCA vasculitis is an autoimmune disease characterized by pathogenic autoantibodies against MPO or proteinase 3 (PR3). MPO and PR3 are abundant in neutrophils and monocytes (8). When ANCAs bind to autoantigens on the surface of activated neutrophils, degranulation is stimulated, which ultimately causes severe small vessel damage (9). In a subset of patients with ANCA vasculitis, disease onset has been linked to the use of drugs, such as hydralazine (10). Although hydralazine is a widely prescribed medication for hypertension and heart failure, the occurrence of associated ANCA vasculitis is rare, but well described.

To understand potential mechanisms of drug-induced autoimmune disease, the pathways of known or potential chemical modifications of the drug in question must be elucidated. Hydralazine is a known carbonyl scavenger that reacts with proteins under oxidizing conditions (11, 12). When neutrophils degranulate in response to events such as microbial infections, released MPO produces reactive oxygen species (ROS), causing cytotoxicity, protein oxidation, DNA damage, and lipid peroxidation. Lipid peroxidation yields molecules such as acrolein (13), a highly reactive unsaturated aldehyde. Through Michael addition, acrolein reacts with an amine group containing amino acids, such as lysine, histidine residues, and the *N*-terminus of proteins. The Michael addition adducts have an aldehyde group that further reacts with nucleophilic groups to generate additional adducts on the protein, which can serve as a neoantigen that triggers an immune response and generation of autoantibodies (14). Among Michael-type addition adducts, *N^ε^*-3-formyl-3,4-dehydropiperidino lysine (FDP-lysine) is a major product (15). The mature form of MPO is a disulfide-linked homodimer that contains multiple residues susceptible to react with acrolein (e.g., 12 Lys, 5 His and 1 *N-*terminus on the heavy chain). It is reasonable to predict that environmentally available acrolein may form carbonyl derivatives with these amino acid residues. Additionally, hydralazine has been shown to react with acrolein-modified and lysine-containing proteins to form hydrazone adducts, which was reported to reduce acrolein toxicity (12).

Our hypothesis posits a sequential process: (a) carbonyl derivatives form on MPO under oxidative conditions (i.e., in the presence of acrolein); (b) hydralazine reacts with these carbonyl derivatives to form hydrazone adducts; and (c) these hydrazone adducts serve as haptens, inducing an autoimmune response through antibody generation. To test this hypothesis, we conducted in vitro studies using commercially prepared myoglobin and MPO to explore chemical reactions, including carbonyl derivatives and hydrazone adduct formation on both proteins. We also explored MPO protein conformational changes after chemical modifications and assessed whether hydrazone adducts could be detected on MPO that was isolated from peripheral blood of patients with hydralazine-associated ANCA vasculitis. We purified IgG and IgM from patients or healthy controls to determine the presence of antibodies specific for hydralazine-modified MPO in individuals with hydralazine-associated ANCA vasculitis. Finally, we observed that antibodies to hydralazine-modified MPO antibodies are pathogenic in an in vivo murine model. Our results suggest that hydralazine modification induces MPO conformational changes and/or hydrazone adduct formation on MPO. This process generates neoantigens or reveals hidden epitopes, which facilitates neoautoantibody generation, thereby leading to the development of ANCA vasculitis.

## Results

### Characteristics of patients involved in the study and people in the healthy control group.

Table 1 provides an overview of the participant cohort characteristics in this study. 5 separate clinicians examined medical records to identify patients with hydralazine-associated vasculitis based on predetermined criteria, which were compared with patients with ANCA vasculitis with no hydralazine exposure. The analysis includes 10 patients diagnosed with hydralazine-associated disease, with a range of hydralazine exposure spanning from 2–57 months prior to presentation. Among this cohort, 60% (*n* = 6) exhibited dual positivity for MPO- and PR3-ANCA, and the other 40% had high-titer MPO-ANCA. Consistent with previous studies (16, 17), all demonstrated high anti-histone antibodies when tested using a commercial histone ELISA (Immuno-Biological Lab). The majority of patients with hydralazine-associated disease had lung and kidney involvement at 60% and 90%, respectively. Conversely, upper respiratory and joint involvement were infrequent (10% and 0%, respectively). Notably, kidney involvement in the hydralazine-associated group was similar to the nonhydralazine-associated patient group, while joint involvement was significantly lower. At the onset of the disease, there were no significant differences between the 2 groups in serum creatinine (2.6 mg/dL versus 2.7 mg/dL) or estimated glomerular filtration rate (eGFR) (23.7 mL/min/1.73m^2^ versus 25.2 mL/min/1.73m^2^). Participants diagnosed with hydralazine-associated disease were significantly older than those without hydralazine-associated disease (72.5 versus 58.4, *P* < 0.006).

### Carbonyl derivative is a prerequisite for hydrazone adduct formation.

Previous studies have shown that hydralazine suppresses acrolein toxicity (12, 18) by forming hydrazones with Michael adducts generated by acrolein (12). We hypothesized that hydrazone adducts form on MPO in patients with hydralazine-associated ANCA. These hydrazone adducts occur through a series of reactions. One prominent adduct formed upon addition of hydralazine to acrolein-modified proteins is a bis-ACR-lysine hydrazone adduct (Supplemental Figure 1; supplemental material available online with this article; https://doi.org/10.1172/JCI178813DS1). Carbonyl modification can be assessed by mass spectrometry or by using 2,4-dinitrophenyl hydrazine (2,4-DNPH), a reagent that reacts with carbonyl groups to produce hydrazones.

**Figure 1:**
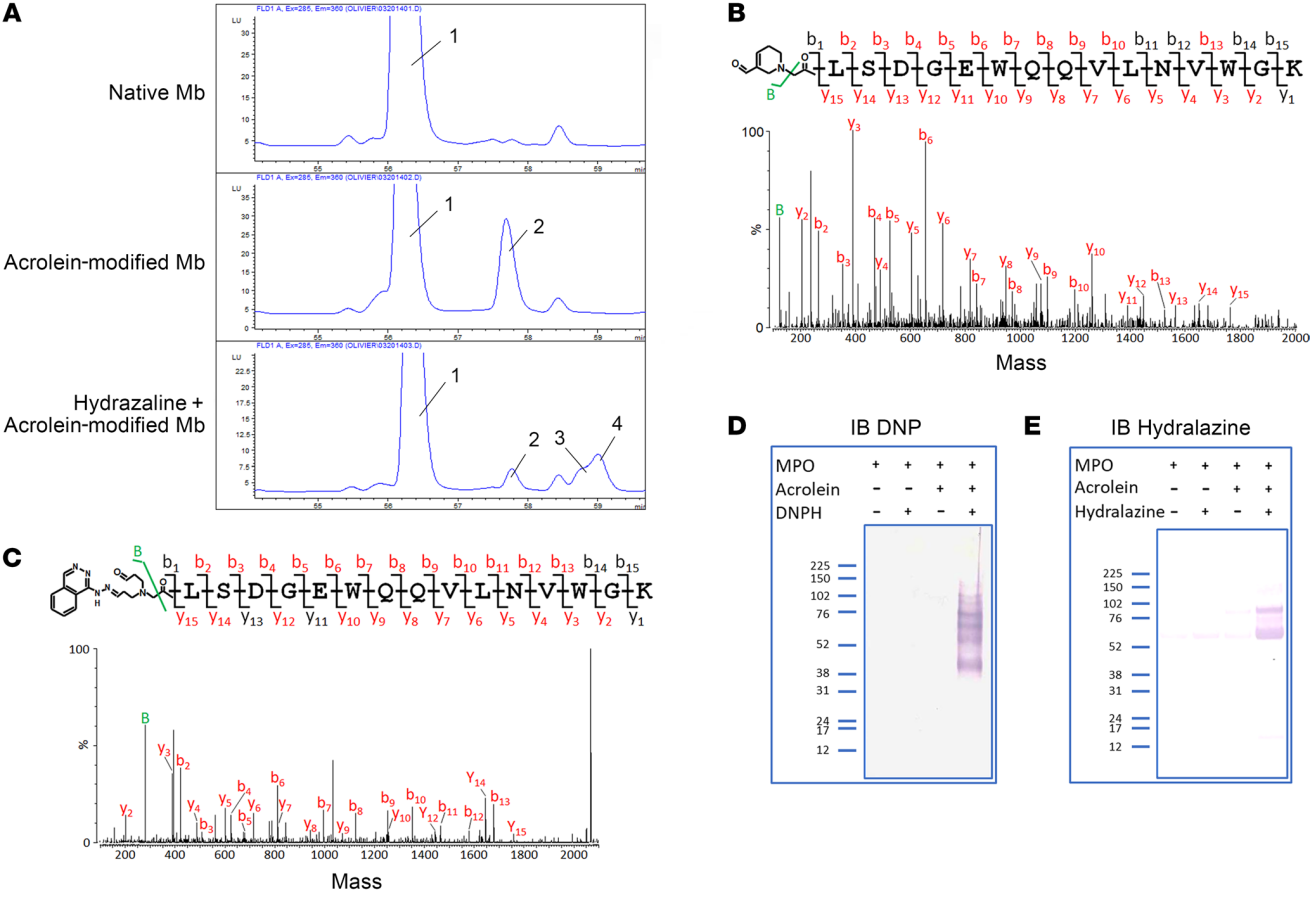
In vitro investigation of carbonyl derivatives and hydrazone adduct formation on myoglobin and MPO. (**A**) C18 RP-HPLC separation of peptides produced by tryptic mapping of purified native myoglobin (Mb), acrolein-modified Mb, and hydralazine + acrolein-modified Mb. Chromatograms showed only the time window in which peptides exhibiting a strong fluorescence at 360 nm (λ_ex_ = 285 nm) eluted. (**B**) Tandem mass spectra (MS/MS) fragmentation spectrum of a FDP derivatized peptide eluting at approximately 57.6 minutes (corresponding to peak 2 of **A**). The deconvoluted tandem mass spectra (MS/MS) spectrum was acquired from a parent ion of m/z 955.50 (+2), which corresponded in mass to *N*-terminal tryptic peptide plus FDP. (**C**) Tandem mass spectra (MS/MS) fragmentation spectrum of a hydrazone-bis-acrolein derivatized peptide eluting at approximately 59.0 minutes (corresponding to peaks 3 and 4 of **A**). The deconvoluted tandem mass spectra (MS/MS) spectrum was acquired from a parent ion of m/z 690.0 (+3), corresponding in mass to tryptic peptide plus a hydrazone-bis-acrolein adduct. (**D** and **E**) Detection of protein-bound carbonyl derivatives and hydrazone adduct by immunoblotting. Intact/unreacted MPO or acrolein-modified MPO were incubated with DNPH (**D**) or with hydralazine (**E**). Protein samples were separated by SDS-PAGE followed by blotting on a nitrocellulose membrane and detection with appropriate antibodies. Each lane contained 2.6 μg of MPO (*n* = 3).

Our initial proof-of-concept experiments were performed using myoglobin. Myoglobin, like MPO, is a heme-containing protein, but is significantly smaller in size (16.9 KDa versus 150 KDa, respectively). Coupled with the size difference and the absence of chemically bound carbohydrates, initial studies with myoglobin facilitated the identification and characterization of modifications on the globin moiety by mass spectrometry. Representative peptide maps with fluorescence detection at 360 nm (λ_ex_ = 285 nm) of native myoglobin (the negative control), acrolein-modified myoglobin, and hydralazine plus acrolein-modified myoglobin are presented in Figure 1A. Comparison of the high-performance liquid chromatography (HPLC) traces revealed several peaks from the digest mixtures of the protein treated with acrolein or with hydralazine plus acrolein that were not present in controls (e.g., peaks 2, 3, and 4). The appearance of peaks 2, 3, and 4 was also accompanied by a significant decrease in intensity of peak 1 relative to control (Figure 1A). HPLC fraction corresponding to chromatographic peaks showing marked variation in fluorescence intensity relative to control were further concentrated and characterized by electrospray ionization mass spectrometry (ESI).

Mass spectrometry analysis of 2 abundant ions of m/z 605.9 (+3) and 908.5 (+2) in peak 1 and 2 abundant ions of m/z 637.3 (+3) and 955.5 (+2) in peak 2, revealed a 94 dalton (Da) increase in mass of acrolein-modified peptide, compared with ions of unmodified peptide, which were consistent with the addition of 1 FDP group to the peptide. The tandem mass spectra (MS/MS) fragmentation spectrum acquired from the ion of m/z 955.5 (+2) is shown in Figure 1B. The spectra showed an almost complete series of both carboxy-terminal y ions (y_2_ to y_15_) and amino-terminal b ions (b_2_ to b_10_ and b_13_). The observed series of b ions showed a 94 Da difference relative to the unmodified *N*-terminal peptide, whereas the masses of the y ions were similar to those observed in the tandem mass spectrometry spectrum of peptide. The mass spectra of peak 3 and 4 exhibited 2 abundant ions of m/z 690.7 (+3) and 1,035.5 (+2) (data not shown), which corresponded in mass to the addition of 254 Da to the unmodified *N*-terminal peptide. The 254 Da mass increase was consistent with the addition of 1 bis-acrolein-hydrazone adduct to the peptide (Figure 1C). Altogether, these data indicate that Gly 1 is the site of formation of the hydralazine-bis acrolein adduct. Because the 2 chromatographic peaks (peaks 3 and 4) showed similar mass spectral data, 2 stereoisomers of the hydrazone adduct were likely formed.

In addition to hydralazine, multiple drugs have been implicated in drug-associated ANCA vasculitis (19). To understand the potential mechanism, we tested additional drugs with similar assays, showing that procainamide and aminoguanidine bound to MPO or myoglobin via carbonyl derivatives in a similar fashion to hydralazine (Supplemental Figure 2, A and B), but propylthiouracil (PTU) bound to myoglobin via sulfenic/sulfinic/sulfonic derivatives (Supplemental Figure 2C). Supporting these observations, aminoguanidine was able to competitively attenuate hydralazine binding to MPO (Supplemental Figure 2D) whereas levamisole, like PTU — bound to MPO via different functional groups — was unable to attenuate hydralazine binding to MPO (Supplemental Figure 2E).

After demonstrating the carbonyl derivative and hydrazone adduct formation on myoglobin, we examined whether the same reactions occurred on MPO in vitro. Using 2,4-DNPH, our in vitro data clearly demonstrated that carbonyl derivatives formed on MPO in the presence of acrolein (Figure 1D). Using an anti-hydralazine antibody, our data showed hydrazone adducts on MPO in the presence of acrolein (Figure 1E). The addition of acrolein was necessary for the formation of these adducts, thus a reactive carbonyl is necessary for hydralazine to react with MPO (Figure 1E).

### Hydrazone adduct formation on MPO induces conformational changes.

**Figure 2:**
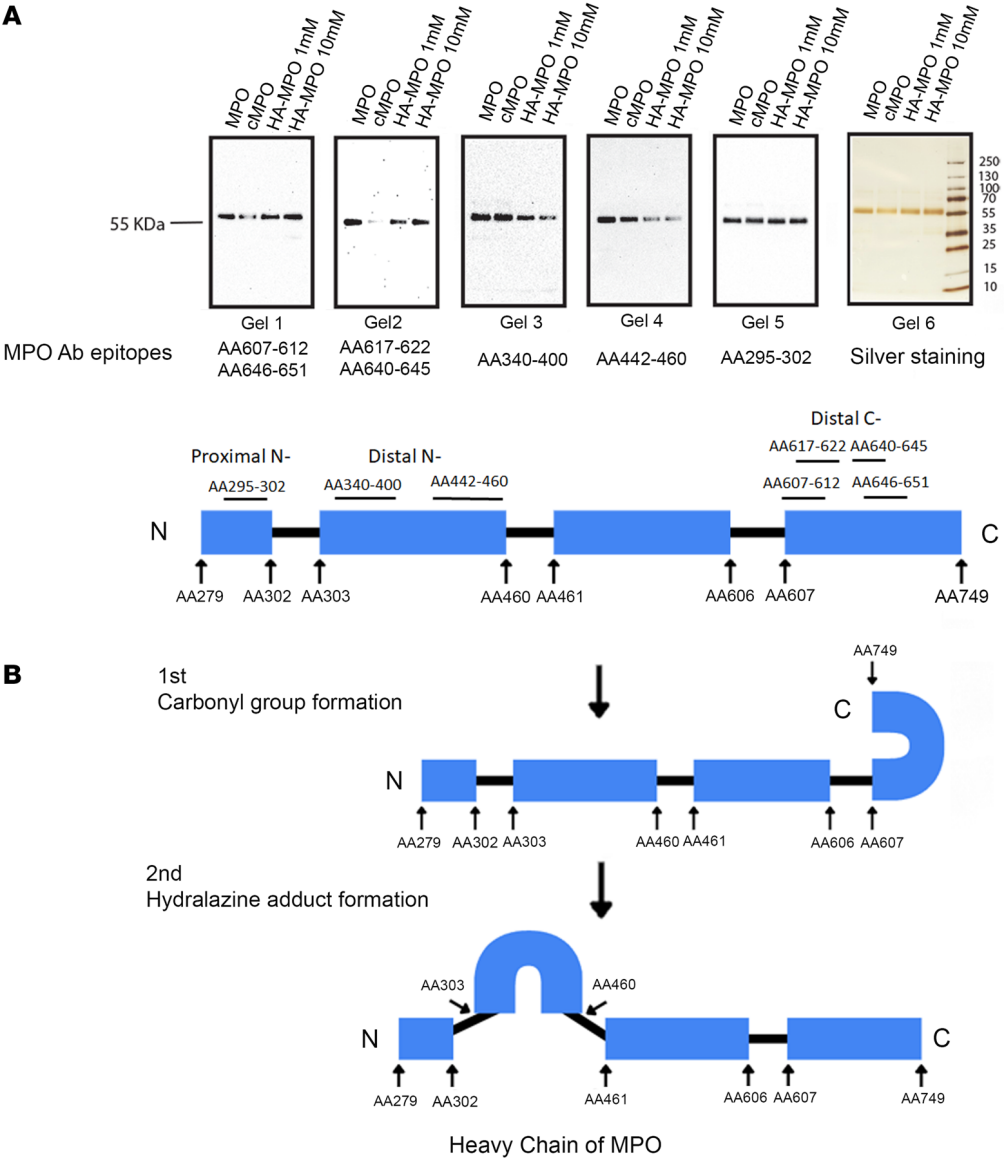
MPO conformational changes after acrolein or acrolein plus hydralazine modification. (**A**) The same amount of native MPO (1 μg), acrolein-modified MPO (cMPO), and 1 mM or 10 mM hydralazine-modified myeloperoxidase (HA-MPO) were loaded and separated by SDS PAGE gels. After transfer to nitrocellulose membranes, the membranes were immunoblotted (IB) with anti-MPO antibodies from Proteintech (gel 1), ABclonal (gel 2), Dako (gel 3), Alpha Diagnostic (custom ordered, gel 4) and R&D system (gel 5), respectively (*n* = 3). The corresponding antibody epitope information was listed under each membrane. Silver staining was performed on a separate membrane (gel 6) for loading control. (**B**) Based on the IB results, a model of MPO heavy chain conformational changes after carbonyl derivative (1st step) and hydrazone adduct formation (2nd step) were proposed.

Following the identification of adduct formation on MPO, our objective was to explore whether these modifications induced conformational changes in MPO. To answer this question, we utilized antibodies specifically targeting various segments of the MPO heavy chain containing potential targets of acrolein. The native MPO heavy chain was uniformly detected by all test antibodies and exhibited comparable sensitivity (Figure 2A). However, the sensitivity of antibodies was more varied in detecting MPO with carbonyl derivatives. Antibodies with epitopes located at the distal C-terminus of the MPO heavy chain, such as the proteintech antibody (hMPO AA607-612, AA646-651; Figure 2A) and ABclonal antibody (hMPO AA617-622, AA640-645; Figure 2A), displayed reduced affinity to MPO with carbonyl derivatives (Figure 2A) (antibody epitope information is based on published epitope mapping data from our lab) (20). Conversely, antibodies with epitopes located at the distal N-terminus of the heavy chain, such as the Dako antibody (hMPO AA340-400, Figure 2A) and anti-KIV antibody (hMPO AA442-460, Figure 2A), or at the proximal N-terminus, such as the R&D System mouse antibody (hMPO AA295-302, Figure 2A) exhibited similar affinity to MPO with carbonyl derivatives compared with native MPO (Figure 2A). These findings suggest that the formation of carbonyl derivatives induced conformational changes of the C-terminus of the MPO heavy chain, which prevented antibody binding in that region (Figure 2B). Hydrazone adducts formed on MPO produced contrasting results regarding conformational changes (Figure 2A). This suggests that the C-terminus of MPO heavy chain reopened (epitopes accessible), while the distal N-terminus of the MPO heavy chain closed (epitopes inaccessible) (Figure 2B). Importantly, these conformational changes in hydralazine-modified MPO were found to be hydralazine dose dependent (Figure 2A). Intriguingly, the proximal N-terminus of the MPO heavy chain remained consistently unchanged under both conditions (Figure 2A).

### Hydrazone adducts on MPO exhibit detectability exclusively in patients with hydralazine-associated ANCA vasculitis.

**Figure 3:**
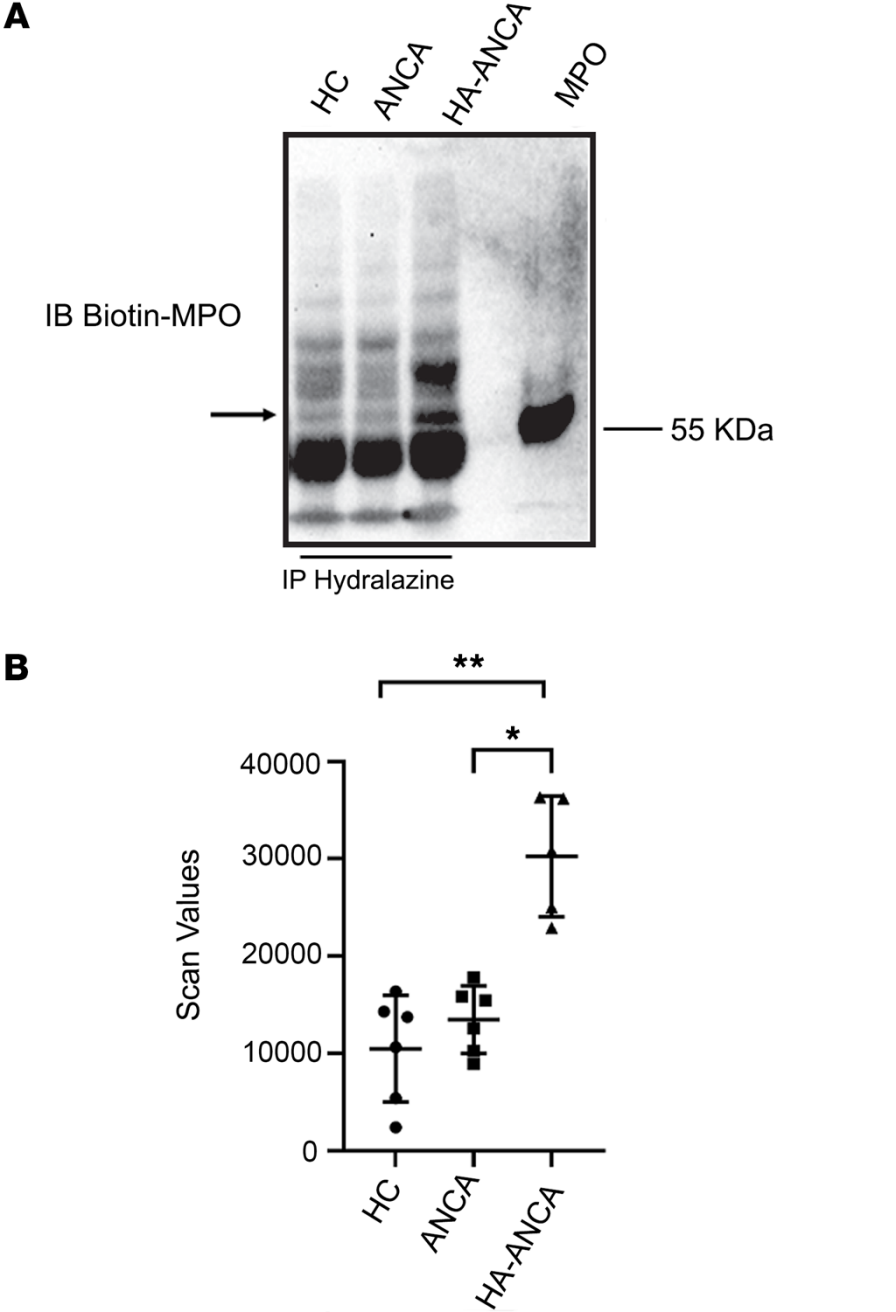
Hydrazone adducts are detected on circulating MPO only from patients with hydralazine associated ANCA. (**A**) Plasmas from healthy participants (HC, *n* = 6 patient samples), patients with nonhydralazine-associated ANCA (ANCA, *n* = 6 patient samples) and patients with hydralazine-associated ANCA (HA-ANCA, *n* = 5 patient samples) were immunoprecipitated with an anti-hydralazine antibody. The immunocomplexes were immobilized with protein G and eluted with DTT containing 2× Laemmli sample buffer before loading SDS-PAGE gels. The separated proteins were transferred to nitrocellulose membrane before immunoblotted with biotinylated anti-MPO antibody. A native MPO was loaded for a positive control. (**B**) The densitometry data were obtained using Image J (NIH, 1.53K version) and the graph was drawn using GraphPad Prism (GraphPad Software, Version 9.5.1). *P < 0.05; **P < 0.01 assessed by Dunn’s multiple comparisons test.

Since all of the above conformational changes were deduced in a controlled in vitro setting, to better elucidate the underlying mechanism of hydralazine-associated ANCA vasculitis, our investigations then focused on the formation of hydrazone adducts on MPO ex vivo. We identified patients with hydralazine-associated ANCA vasculitis, nonhydralazine-associated ANCA vasculitis with high-titer MPO-ANCA serologies, and people who were in a healthy control group. Our hypothesis suggests the presence of hydralazine protein adducts in the circulation. Therefore, we employed immunoprecipitation with an anti-hydralazine antibody to enrich proteins containing hydrazone adducts. Subsequently, we utilized a biotinylated anti-MPO antibody to assess the formation of hydrazone adducts on MPO ex vivo with native MPO serving as a control. Our findings revealed the detection of hydrazone adducts on MPO (heavy chain) exclusively in the patients with hydralazine-associated ANCA vasculitis. In contrast, individuals with no known hydralazine exposure or healthy participants exhibited minimal MPO reactivity, indicating an absence of hydrazone adducts on circulating MPO (Figure 3, A and B). Remarkably, a patient with nonhydralazine-associated ANCA vasculitis, initially misclassified with the hydralazine-associated cohort, did not exhibit MPO reactivity in the same assay (Supplemental Figure 4). This study marks the demonstration of hydrazone formation on MPO ex vivo. To confirm the ex vivo formation of hydrazone adducts on protein, we examined circulating histones since previous studies (5,6) showed most patients with hydralazine-associated ANCA vasculitis were also anti-histone–antibody positive. Consistently, histone protein was detected among the proteins enriched for hydrazone adducts exclusively in patients with hydralazine-associated ANCA vasculitis (Supplemental Figure 3).

### IgM is the primary immunoglobin against hydrazine-modified MPO.

**Figure 4:**
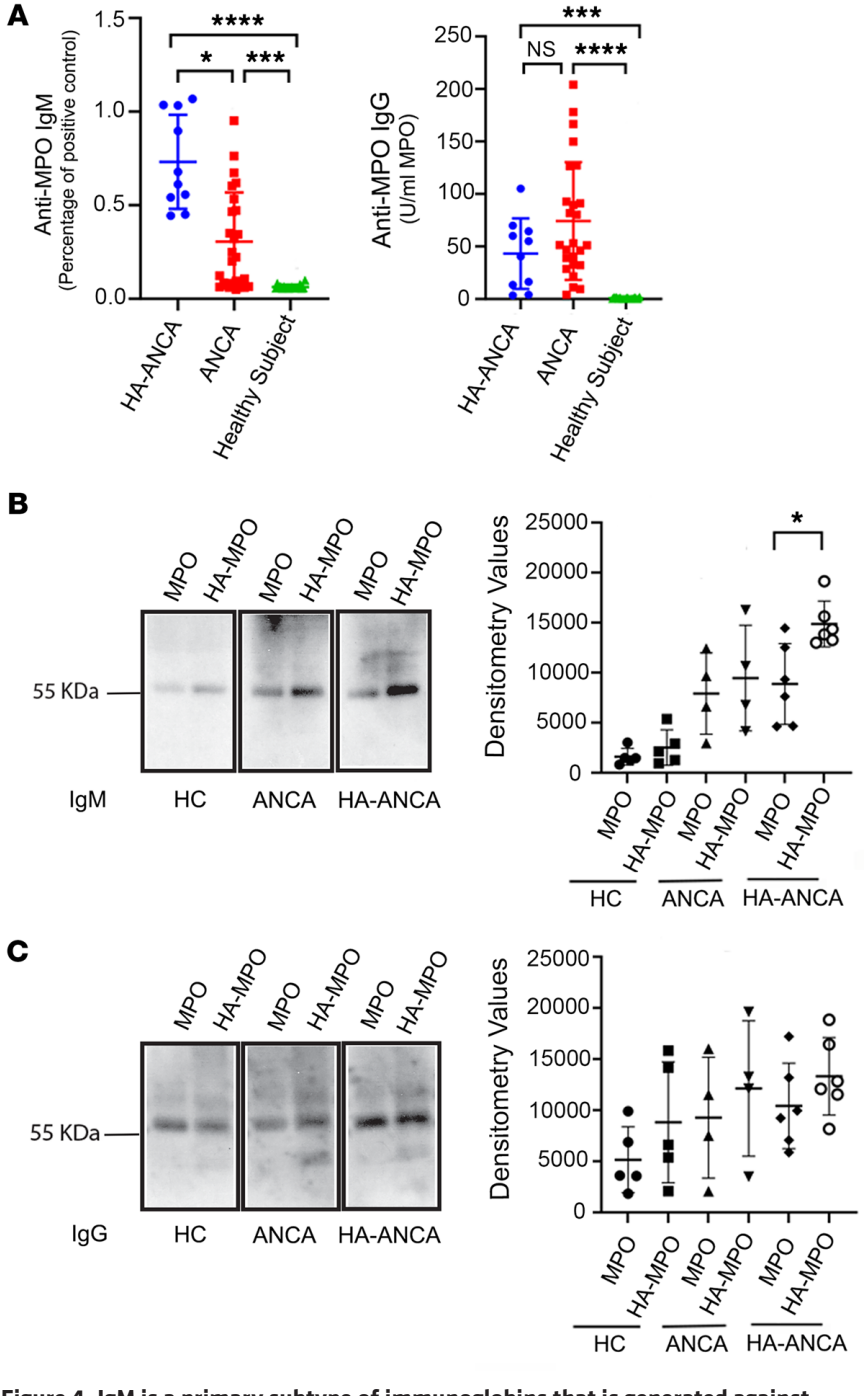
IgM is a primary subtype of immunoglobins that is generated against hydralazine-modified MPO ex vivo. (**A**) Plasma anti-myeloperoxidase IgG and IgM from patients with hydralazine-associated ANCA (*n* = 10), patients with nonhydralazine-associated ANCA (*n* = 25), and participants in a healthy control group (*n* = 19) were measured following the protocol described in the Methods section. The multiple comparisons of IgM or IgG for any 2 groups of patients’ *P* values (*P < 0.05; ***P < 0.001; ****P < 0.0001) were calculated by Dunn’s multiple comparisons test. (**B** and **C**) IgG and IgM were purified from plasma obtained from healthy participants (HC, *n* = 5), patients with nonhydralazine-associated ANCA (ANCA, *n* = 4), and patients with hydralazine-associated ANCA (HA-ANCA, *n* = 6). The same amount of native MPO and hydralazine-modified MPO were separated using SDS-PAGE gels and transferred to nitrocellulose membranes. The membranes were immunoblotted with IgM (**B**) and IgG (**C**). Densitometry values were obtained using Image J (NIH, 1.53K version) and graph was drawn using GraphPad Prism (GraphPad Software, Version 9.5.1). *P < 0.05 assessed by Mann-Whitney test for comparison between 2 groups.

**Figure 5:**
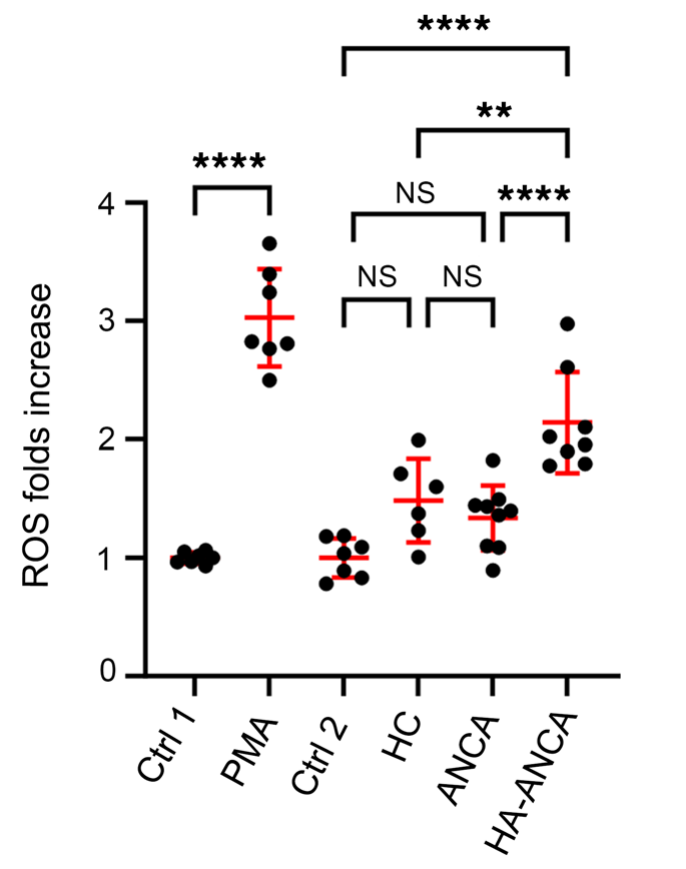
IgM purified from patients with hydralazine-associated ANCA significantly induces oxidative burst in neutrophil-like HL-60 cells. Differentiated (neutrophils-like) HL-60 cells (1 × 10^5^) were seeded to each well of a 96 well plate. Cytochalasin B (2 μM) was used to prime the cells before adding each treatment, including PMA (100 ng/mL), IgM (0.75 μg/well) isolated from individuals who were healthy controls (HC, *n* = 6), patients with nonhydralazine-associated ANCA (ANCA, *n* = 9),or patietns with hydralazine-associated ANCA (HA-ANCA, *n* = 8). Reactive oxygen species (ROS) was detected by DHE (2 μM) and the plate was read by a TECAN with EX 510 nm/EM 610 nm. Since the peak value for PMA and IgM treatment appeared at separate time points, control 1 (Ctrl 1) was control value for PMA treatment and control 2 (Ctrl 2) was control value for IgM treatment. Graphs were drawn using GraphPad Prism (GraphPad Software, Version 10.1.0) **P < 0.01; ****P < 0.0001 assessed by 1-way ANOVA multiple comparisons test.

Drawing on previous research demonstrating that an acrolein-lysine adduct can constitute an antibody epitope (12), we postulated that hydrazone adducts on MPO could similarly act as a hapten and elicit an autoimmune response in vivo. To test this theory, we measured the levels of anti-MPO IgG and IgM in our patient cohort. Measurement of anti-MPO IgG and IgM in plasma supported this hypothesis, revealing significantly higher levels of anti-MPO IgG in patients with both hydralazine- and nonhydralazine-associated ANCA compared with people who were in a healthy control group (Figure 4A). When examining the IgM subclass, both patient groups had higher anti-MPO IgM levels than healthy participants; however, the median level of anti-MPO IgM in hydralazine-associated patients was significantly higher than patients with nonhydralazine-associated ANCA (0.65 (0.54, 1.03) versus 0.06 (0.06,0.07), *P* < 0.0001) (Figure 4A). This suggests that the IgM subclass of ANCA may be a key immunoglobulin isoform resulting from hydralazine exposure and formation of neoantigen. Isolation of IgM and IgG from each patient group confirmed this hypothesis. IgM from hydralazine-associated ANCA vasculitis patients exhibited robust reactivity against hydralazine-modified MPO, consistent with ELISA results, indicating IgM as the main antibody isoform that can specifically react with hydralazine-modified MPO (Figure 4B). In contrast, IgG from patients with hydralazine-associated ANCA vasculitis showed similar affinity to both types of MPO (Figure 4C). Notably, no significant difference in IgG binding to native MPO was detected between patients with nonhydralazine-associated ANCA and healthy participants, although a trend of increased binding was detected in patients with ANCA (Figure 4C). In addition, IgM and IgG isolated from patients with nonhydralazine-associated ANCA vasculitis displayed similar affinity with both hydralazine-modified MPO and native MPO (Figure 4, B and C). To confirm these key results, a direct binding assay was performed. Consistent with Western blots results, IgM purified from patients with hydralazine-associated ANCA vasculitis had significantly higher binding to hydralazine-modified MPO than to native MPO. IgM purified from patients with nonhydralazine-associated ANCA had very low binding ability to both types of MPO (Supplemental Figure 5A). In addition, native MPO binding by IgG from patients with nonhydralazine-associated ANCA was significantly higher than that of IgG from people in a healthy control group (Supplemental Figure 5B). We also examined the ability of purified IgM from each group to induce degranulate neutrophils. Neutrophil-like HL-60 cells were treated with IgM purified from each group of patients and healthy controls. The results clearly showed that IgM isolated from patients with hydralazine-associated ANCA had the strongest ability to stimulate ROS generation while IgM purified from healthy participants and patients with nonhydralazine associated ANCA had no difference in their effect on ROS generation compared with controls (Figure 5). All these data suggest that IgM is the primary type of immunoglobin against hydralazine-modified MPO and may be pathogenic.

### Hydralazine-modified MPO induces glomerulonephritis in anti-MPO splenocyte-recipient mice.

**Figure 6:**
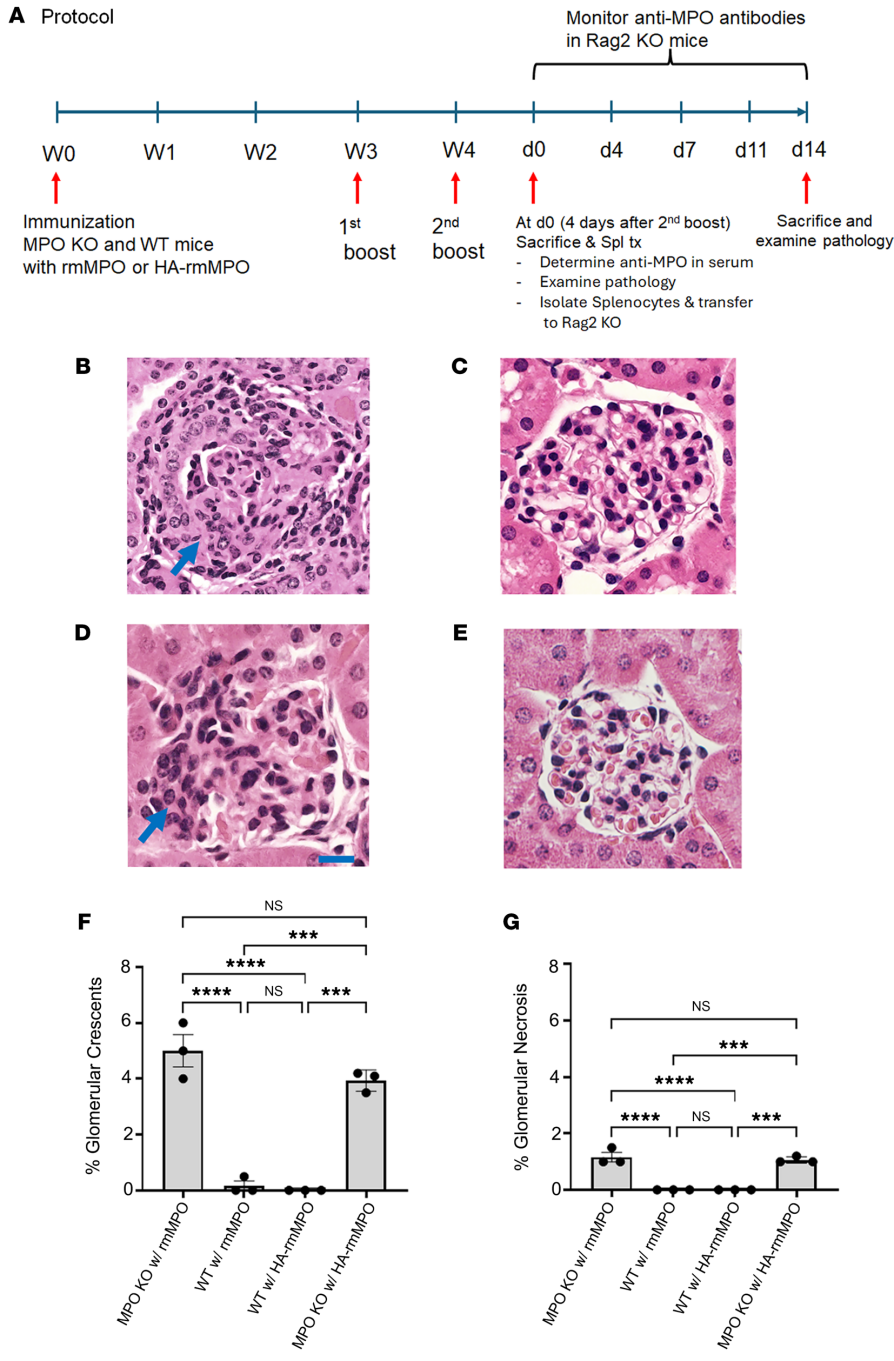
Glomerulonephritis is induced in the Rag2-knockout mice by transfer of splenocytes from MPO-knockout mice immunized with hydralazine-modified recombinant mouse MPO. (**A**) Schematic of splenocyte transfer model. (**B**–**E**) Splenocytes from MPO^–/–^ mice immunized with recombinant mouse MPO (rmMPO) (**B**, *n* = 3) or HA-rmMPO (**D**, *n* = 3) caused glomerulonephritis, indicated by crescent formation (arrow) (scale bar: 20 μm). Splenocytes from WT mice immunized with rmMPO (**C**, *n* = 3) or HA-rmMPO (**E**, *n* = 3) did not cause glomerulonephritis in Rag2-knockout mice after transfer. (**F**) Quantification of glomerular crescents. (**G**) Quantification of glomerular necrosis. Data are expressed as mean ± SEM. Graph was drawn using GraphPad Prism (GraphPad Software, Version 9.5.1). ***P < 0.001; ****P < 0.0001 assessed by 1-way ANOVA with multiple comparisons.

Based on our in vitro and ex vivo data demonstrating the association of hydralazine-modified MPO with development of ANCA-vasculitis, we sought direct evidence that hydralazine-modified MPO is pathogenic. Rag2^−/−^ mice were used to test the pathogenicity of antibodies produced by transplantation of splenocytes from MPO^–/–^ mice immunized with hydralazine-modified recombinant mouse MPO (HA-rmMPO) or unmodified rmMPO. Four days after the second boost with HA-rmMPO, splenocytes isolated from HA-rmMPO–immunized MPO^–/–^ mice were transferred to Rag2^–/–^–recipient mice. After 2 weeks, the mice were sacrificed, and kidney sections were examined histologically for glomerular necrosis and crescent formation (Figure 6A). As positive and negative controls, unmodified rmMPO was also used to immunize MPO^–/–^ mice and WT mice. H&E staining of kidney sections clearly showed glomerulonephritis from splenocytes derived from rmMPO immunized MPO^–/–^ mice (Figure 6B) but not from splenocytes derived from immunized WT mice (Figure 6C). Importantly, splenocytes from HA-rmMPO–immunized MPO^–/–^ mice (Figure 6D) but not WT mice (Figure 6E) also induced glomerulonephritis. Glomerular crescents were quantified for each group (Figure 6F) with a similar pattern of glomerular necrosis (Figure 6G). In addition, like unmodified MPO, mice immunized with HA-rmMPO generated antibody (IgG but not IgM) only in MPO^–/–^ mice but not in WT mice (Supplemental Figure 6, A–D). Similarly, splenocytes from unmodified rmMPO or hydralazine-modified rmMPO–immunized MPO^–/–^ mice generated antibody (IgG only) in recipient Rag2^–/–^ mice, but neither induced antibodies in WT mice (Supplemental Figure 7, A–D).

### T cells from hydralazine-modified MPO–immunized mice preferentially secrete IL-17A.

To determine the contribution of T cells to the anti-hydralazine-modified MPO immune response, total splenocytes were harvested from both WT and MPO^–/–^ mice immunized with either unmodified rmMPO or HA-rmMPO. ELISpots for both IFN-γ and IL-17A were utilized to determine specific T cell responses to either antigen (unmodified rmMPO or HA-rmMPO). The T cell IFN-γ response was more robust in response to native rmMPO compared with HA-rmMPO (Supplemental Figure 8, A and B). However, T cell IL-17A release was similar in response to native rmMPO and HA-rmMPO (Supplemental Figure 8, C and D). Interestingly, little to no IL-17A release was detected from T cells from WT mice immunized with either antigen; only MPO^–/–^ mice immunized with either antigen produced IL-17A in response to their respective immunized antigen (Supplemental Figure 8, C and D). As IL-17A is highly linked to development of autoimmune disease, T cell production of IL-17A in response to both unmodified rmMPO and HA-rmMPO supports the assessed development of glomerulonephritis in mice receiving splenocytes from immunized mice. Importantly, unlike unmodified rmMPO, HA-rmMPO only stimulated an IL-17A response, indicating that its pathogenic mechanism may be different from unmodified rmMPO.

## Discussion

Drug-associated autoimmune diseases, including drug-associated ANCA vasculitis, have been reported, but the underlying mechanisms are unclear (6, 21). We describe a pathogenic mechanism to explain drug-induced autoimmunity within the context of hydralazine-associated ANCA vasculitis. We found that oxidative conditions facilitate hydrazone adduct formation on MPO in vitro and on circulating MPO in patients with hydralazine-associated ANCA vasculitis (ex vivo). These patients had MPO-ANCA of the IgM subtype with high affinity for hydralazine-modified MPO. We also found that patients with hydralazine-associated disease had elevated IgM titers against unmodified MPO compared with patients with nonhydralazine-associated disease or healthy participants. Additionally, our in vitro data demonstrated that hydrazone adduct formation on MPO resulted in MPO protein conformational changes, which may further explain the autoimmune response in vivo. These findings implicate MPO-hydrazone adducts and MPO conformational changes as a causative mechanism for the development of hydralazine-associated ANCA vasculitis.

Consistent with other studies (16, 17), all patients with hydralazine-associated ANCA vasculitis in this study were seropositive for MPO-ANCA and/or exhibited dual seropositivity for both MPO-ANCA and PR3-ANCA. Therefore, our study concentrated on the modifications of MPO following exposure to hydralazine, particularly in the context of acrolein. We initiated our investigation by examining the conditions of hydrazone adduct formation on MPO in vitro. Previous studies demonstrated acrolein’s ability to modify lysine and histidine residues of human serum albumin (22) and α-1-proteinase (23), resulting in the formation of carbonyl derivatives. Additionally, research has shown that hydralazine effectively traps acrolein adducts in proteins, mitigating acrolein-mediated toxicity (12, 18). Utilizing a lysine-containing peptide, previous studies demonstrated hydrazone adduct formation in the presence of hydralazine via Michael adducts generated by acrolein (12). This led us to hypothesize that the formation of carbonyl derivatives may be necessary for hydrazone adduct formation on a target protein. Our data align with this hypothesis, suggesting that carbonyl derivatives generated by acrolein are a prerequisite for hydrazone adduct formation when a protein is exposed to hydralazine.

A previous study showed the importance of the oxidation step through the formation of nicotinamide adenine dinucleotide phosphate–dependent (NADPH-dependent) dihydralazine metabolites that reacted with heme to bind microsomal proteins (24). Consistently, our data suggest that carbonyl derivative generated by acrolein was prerequisite for hydrazone adduct formation when a protein was exposed to hydralazine. In conjunction with our data, this confirms our hypothesis that carbonyl derivative formation is a key step in the development of hydralazine-associated ANCA vasculitis. The variability in carbonyl adduct formation among patients exposed to hydralazine could explain why not all patients exposed to hydralazine develop ANCA vasculitis. We hypothesize that additional triggers, such as high-pollutant exposure (25), diets with high temperature, oily foods (26), or oxidative stress exposures (27), may contribute to the development of hydralazine-associated vasculitis. In addition, multiple drugs have been reported to associate with ANCA vasculitis (19). Our study demonstrates that the mechanism that mediates drug binding to MPO depends on its functional group. For example, hydralazine, procainamide, or aminoguanidine, which contains amine group, bind to MPO via carbonyl derivatives. While PTU or levamisole binds to MPO via sulfenic/sulfinic/sulfonic derivatives. More pointedly, early clinical trials of aminoguanidine to prevent microvascular disease in patients with diabetes were complicated by several cases of overt MPO antibody associated ANCA vasculitis, which presented as crescentic glomerulonephritis (28). This effect also appeared to be dose dependent, as no participants in the low-dose arm of the study developed this complication.

We observed conformational changes in the autoantigen MPO after carbonyl or hydrazone adduct formation (Figure 2). Previous research identified structural alterations in MPO-inactivated peroxidase activity after propylthiouracil administration in rats (29). Our study appears to be the first to associate such changes with a known drug driving conformational changes in MPO that can potentially trigger the autoimmune response by unveiling cryptic epitopes. Recognizing the critical role of a protein’s conformational structure in its immunogenicity (30), our lab has shown that MPO epitope antigenicity was conformational in 20 of 25 epitopes (31). Our current investigation revealed that carbonyl derivatives and hydrazone adducts induced distinct conformational changes in MPO. These changes may result in the exposure of different epitopes, leading to the generation of neoautoantibodies — some pathogenic and others nonpathogenic. This variability may also explain why not all hydralazine-exposed patients develop ANCA vasculitis.

Of note, when using an antibody specific to a well-known pathogenic MPO epitope (AA 442–460) (31), we observed significantly reduced reactivity to hydralazine-modified MPO (Figure 2A, gel 4). This suggests that other epitopes may be responsible for hydralazine-associated ANCA vasculitis. In addition, we also demonstrated that both carbonyl derivative and hydrazone adduct formation led to C-terminus conformational changes in the heavy chain of MPO, indicating the importance of this region. Interestingly, one of our early MPO epitope studies revealed that MPO-ANCA mainly targeted the C-terminus of the heavy chain of MPO (32). Later, using overlapping decapeptides to represent the MPO protein, 5 of 7 major epitopes were found on the heavy chain of mature MPO, one located within the light chain of MPO, and another located within the propeptide region of the protein (33). In general, MPO epitopes are highly variable among patients and not all autoantigen epitopes are pathogenic. Indeed, it has been shown that almost 90% of patients exposed to procainamide, one of the most common drugs leading to drug-induced lupus, developed a detectable anti-nuclear antibody while only 30% of them developed clinical features of lupus (34). Our current data indicate that potential epitopes in patients with hydralazine-associated ANCA vasculitis may be located within the C-terminus or the N-terminus of the MPO heavy chain.

The induction of autoimmunity via haptenation, triggered by chemical and drug exposure, has been documented in previous research (35). For example, an in vitro study revealed that the metabolism of dihydralazine by P450 1A2 produces reactive metabolites, leading to immunoallergic hepatitis characterized by anti-cytochrome P450 1A2 autoantibodies (24). Given that hydrazone adducts and conformational changes in hydralazine-modified MPO may act as neoantigens in vivo, our investigation delved into the potential corresponding autoantibodies. Notably, we identified antibodies reacting to both hydralazine-modified MPO and native MPO in patients, with these antibodies belonging to the IgM and IgG subclasses. Interestingly, the affinity for hydralazine-modified MPO was more prominent in the IgM subclass. A previous study indicated that circulating IgM isotype antibodies against MPO were present during the initial months in 9 patients with hydralazine-associated ANCA vasculitis, followed by an increase in the IgG isotype later (36). In addition, a recent study demonstrated that IgM-dominant immune complex–mediated glomerulonephritis has been associated with hydralazine associated positive ANCA and anti-nuclear antibody (37). Furthermore, elevated IgM levels manifested in specific autoimmune diseases, indicating that IgM regulates autoimmunity (38). Indeed, IgM autoantibodies have been shown to contribute significantly to the pathogenesis of various autoimmune diseases, such as rheumatoid arthritis, autoimmune hemolytic anemia, and autoimmune neuropathy (39). Our study further supports this concept and shows that IgM autoantibodies are responsible for pathogenesis in hydralazine-associated ANCA vasculitis. Therefore, the IgM signature revealed in our current study could serve as a biomarker for patients with drug-associated ANCA vasculitis.

Herein, our data provides a mechanism for hydralazine-associated ANCA vasculitis. However, our focus on heavy chain conformational changes after hydralazine exposure neglects the potential importance of MPO light chain reactivity as shown in previous studies of patients with hydralazine-associated ANCA vasculitis (33, 40). Further investigations should examine the MPO light chain, particularly considering its association with more severe disease status (40) and the light chain of MPO also contains several amine groups containing amino acids that can potentially form carbonyl derivatives and hydrazone adducts. In addition, this study was unable to detect carbonyl modification in vivo because of the limitation of current technology and animal models to separate 2 step-wise reactions, which may occur simultaneously or quickly in vivo. Furthermore, it is not currently feasible to exclusively isolate the antibodies from patients that only recognize hydralazine-modified MPO but not native MPO, which may explain why the IgG isolated from patients with nonhydralazine-associated ANCA was able to minimally recognize hydrazone MPO. In future study, we plan to work on epitope mapping using IgG and IgM isolated from patients with hydralazine-associated ANCA to determine if unique epitopes are responsible for the onset of hydralazine-associated ANCA vasculitis.

In summary, we propose a mechanism for the formation of hydrazone adducts on MPO based on in vitro and ex vivo data. Our experiments strongly support our hypothesis that the development of hydralazine-associated ANCA is dependent on the sequential events of carbonyl derivatives and hydrazone adducts formation on MPO. Moreover, we demonstrated that carbonyl derivatives and hydrazone adduct fundamentally altered the conformational structure of MPO in vitro, with corresponding autoantibodies detected in samples from carefully selected patients with hydralazine-associated ANCA vasculitis. We also observed that the IgM subclass isolated from patients with hydralazine-associated ANCA vasculitis exhibited higher reactivity to hydralazine-modified MPO. This finding provides strong evidence that hydrazone adduct formation on MPO (serving as a hapten) and/or MPO conformational alterations (serving as neoantigens) may elicit autoantibody generation. Importantly, the pathogenicity of hydralazine-modified MPO was confirmed in an animal model. Our study provides a framework for understanding the pathogenesis of other drug-associated autoimmune diseases and has the potential to illuminate the mechanisms of autoimmunity in general. For patients and clinicians, our observations set the groundwork for future diagnostic tool development to rapidly identify cases of hydralazine-associated ANCA vasculitis.

## Methods

### Sex as a biological variable

For human studies, samples from both men and women (Table 1) were used. No sex-specific differences were found. For animals, our previous study (41) demonstrated that there was no sex difference in the MPO immunization and adoptive splenocyte transfer model. In this study, we used male mice (WT, MPO^–/–^ and Rag2^–/–^) only. Of note, same-sex mice must be used for splenocyte transfer, as transfer between different-sex mice resulted in lower antibody production than transfer between the same-sex mice.

### Human subjects

Patients diagnosed with ANCA small-vessel vasculitis were classified according to the Chapel Hill Consensus Conference nomenclature (42) and identified through the Glomerular Disease Collaborative Network (GDCN) (43). Blood samples were collected in accordance with GDCN-established protocols during patient visits for routine clinical care.

To ensure accuracy, 5 physicians independently reviewed patient medical records to confirm disease status and assess any association with hydralazine. Patients were categorized as having hydralazine-associated ANCA if they met specific criteria: (a) documented evidence of hydralazine treatment for a clinically significant period prior to disease onset (e.g., documented in outpatient progress notes or as chronic home medication upon admission) and (b) presented with an elevated anti-MPO antibody or (c) dual anti-MPO and anti-PR3 antibodies.

Exclusions were made for patients with exposure to other common causes of drug-induced vasculitis (e.g., levamisole) or those with hydralazine exposure only after the onset of symptoms (e.g., administered while admitted for new diagnosis of vasculitis). Given previous findings indicating that hydralazine-associated ANCA vasculitis patients are primarily MPO positive (or dual positive), our study focused on including MPO-positive, nonhydralazine comparators.

Disease onset was defined as the initiation of clinical symptoms based on chart review and/or histopathological diagnosis. ANCA serotypes were determined through antigen specific PR3 or MPO ELISA, as described previously (44).

### Materials

MPO from human neutrophils (lyophilized, more than 1,000 units/mg) was obtained from Lee Biosolutions and MPO isolated from leukocytes of purulent human sputum obtained from Elastin Products Company (Cat MY862). Active recombinant mouse MPO (Cat MPO-428M) was purchased from Creative BioMart Inc. Horse heart myoglobin (lyophilized) was obtained from US Biological. Trypsin (from porcine pancreas, modified, sequencing grade) was obtained from Promega. Acrolein diethylacetal and protein G agarose (Cat 16-266) were obtained from Sigma-Aldrich. EZ-link Sulfo-NHS-LC-Biotin (Cat A39257), Streptavidin Agarose Resin (Cat 20353), human IgG ELISA kit (Cat BMS2091) and human IgM ELISA kit (Cat 2098) were obtained from Thermo Fisher Scientific. MPO IgG ELISA plate kit was obtained from Inova Diagnostics (Cat 704655). Commercial anti-MPO antibodies were obtained from DAKO (Cat A0398; Agilent Technologies), Proteintech (Cat 22225-1-AP), ABclonal (Cat A1374), and R&D systems (Cat MAB3174). In addition, anti-MPO (KIV) (AA441-460, CLYQEARKIVGAMVQIITYR as an immunogen) antibody was custom ordered from Alpha Diagnostic Intl. Inc. Anti-hydralazine antibody was obtained from antibodies.com (Cat ABIN343204). All other chemicals were of analytical grade and were obtained from Sigma-Aldrich or Roche Molecular Biochemicals.

### Preparation and purification of acrolein-modified myoglobin and hydralazine-modified myoglobin in vitro

Reactions were conducted at 37°C in 100 mM potassium phosphate buffer (pH 7.4) containing 500 μM myoglobin and 2 mM acrolein in 0.1 M hydrochloric acid (HCl). After a 2-hour incubation, samples were loaded onto a PD-10 gel filtration column and eluted with 100 mM potassium phosphate buffer (pH 7.4) to remove unreacted acrolein. The PD-10–purified fractions having the highest myoglobin content were collected, pooled, cooled to 4°C, and acidified to pH 2.5 by the addition of concentrated HCl. The heme was then extracted with 1 volume of cold 2-butanone. The organic layer containing the extracted heme was discarded. The aqueous solution containing the apoprotein was loaded on a PD10 column and eluted with 50 mM NH_4_HCO_3_ buffer (pH 7.8). The fractions having the highest myoglobin content were collected, pooled, and digested by trypsin, as described in the protein digestion section.

Solutions of intact/unreacted myoglobin (control) and acrolein-modified myoglobin, prepared as above, were added to an equal volume of hydralazine (hydralazine hydrochloride; 10 mM in H_2_O). After a 1-hour-and-30-minute incubation at 37°C, unreacted hydralazine was removed from the samples by passage through a PD-10 column preequilibrated and eluted with 100 mM potassium phosphate buffer (pH 7.4). The fraction having the highest myoglobin content was collected, cooled to 4°C, and the heme was extracted with 1 volume of cold 2-butanone, as described above. The aqueous solution containing the apoprotein was loaded onto a PD-10 column and eluted with 50 mM NH_4_HCO_3_ buffer (pH 7.8). The fractions having the highest myoglobin content were pooled.

### Myoglobin protein digestion

Samples of intact/unreacted myoglobin (control), acrolein-modified myoglobin, and hydralazine + acrolein-modified myoglobin, prepared as above, were diluted in the digestion buffer (50 mM NH_4_HCO_3_, pH 7.8) to a final concentration of approximately 10 μM and digested using a 64:1 (w/w) substrate-to-trypsin ratio for 16 hours at 37°C. Reactions were stopped by injecting the final mixtures directly onto the C18 reverse-phase high-performance liquid chromatography (RP-HPLC) column, which was operated as described in the HPLC analyses section.

### Fractionation of myoglobin protein digest by RP-HPLC

All RP-HPLC experiments were performed on a Chemstation 1100 liquid chromatography system (Agilent Technologies), equipped with diodearray UV-visible and fluorescence detectors. Digested samples (200 μL) were injected onto a Vydac 218TP54, 4.6 mm × 250 mm, C18 RP-HPLC column (Vydac). Peptides were eluted using a flow rate of 0.8 mL/min and a linear gradient rising from 100% solvent A (0.1% trifluoroacetic acid in water) to 50% solvent B (0.085% trifluoroacetic acid in acetonitrile) in 80 minutes. HPLC fractions were collected with a fraction collector. Fractions containing acrolein-modified and hydralazine plus acrolein-modified peptides were pooled, lyophilized, and stored at −70°C for subsequent analysis.

### Electrospray mass spectrometry

ESI and MS/MS were acquired with a Micromass Q-TOF Micro (Waters Micromass Corporation) hybrid tandem mass spectrometer. All experiments were performed in the positive ion mode. Lyophilized HPLC fractions of acrolein-modified, and hydralazine + acrolein-modified peptides were resuspended in a minimal volume (approximately 50 μL) of 50:50 water:acetonitrile containing 0.1% formic acid and infused into the mass spectrometer at approximately 300 nL/minute using a pressure injection vessel. The instrumental parameters were as follows: capillary voltage, approximately 3,300 V; sample cone voltage, 40V; collision energy, between 10 and 50 eV; source temperature, 80°C. Data analysis was accomplished with a MassLynx data system and MaxEnt deconvolution software supplied by the manufacturer.

### Hydrazone adduct formation on MPO in vitro

Purified MPO (4 μM) was first incubated with 2 mM acrolein and then the indicated concentration of hydralazine was added for overnight incubation at 37°C. Following the reaction, MPO that reacted with or without hydralazine was separated on SDS-PAGE gels under reducing condition. The membrane was incubated overnight at 4°C with rabbit anti-human MPO antibody (Dako, A0398) at a 1:4,000 dilution or sheep anti-hydralazine antibody (antibodies-online.com ABIN343204) at a 1:200 dilution.

### Immunoprecipitation and immunoblotting hydrazone-adducted MPO

Immunoprecipitation was performed by incubating 60 μL of plasma with 3 μL of anti-hydralazine antibody at 4°C overnight. The immunocomplexes were immobilized using protein G agarose beads for 2 hours at 4°C and washed 3 times with PBS. The precipitated proteins were eluted in 40 μL of 2 × Laemmli sample buffer, boiled for 5 minutes, and separated using a 7.5% SDS-PAGE gel. The proteins were transferred to Immobilon-P membranes that were blocked for 1 hour in 5% nonfat milk in Tris-saline buffer with 0.2% Tween 20. The blots were incubated overnight at 4°C with biotinylated anti-MPO antibody (1:250 dilution). The proteins were visualized using enhanced chemiluminescence (Thermo Fisher Scientific).

### MPO heavy chain conformational changes

The same amounts of native MPO, carbonyl-derivative MPO, and 2 different concentrations of hydralazine-modified MPO (1 mM and 10 mM) were loaded and separated on SDS-PAGE gels and transferred to PVDF membranes. The membranes were immunoblotted with different anti-MPO antibodies generated by different antigens.

### IgM and IgG purification from plasma

Anti-human IgM antibody was biotinylated following manufacturer protocol. Each plasma sample was incubated with biotin-anti-IgM antibody overnight at 4°C and immobilized with streptavidin beads. Beads were washed 3 times with PBS before eluting with 0.1 M glycine (pH 2.4) and neutralizing with Tris-buffer (pH 9.0) immediately. After removing IgM, the remaining plasma was incubated with protein G agarose beads overnight at 4°C and IgG was eluted with 0.1 M glycine (pH 2.4) followed by neutralization with Tris-buffer (pH 9.0). Purities of IgM and IgG were examined with SDS-PAGE gels (Supplemental Figure 9).

### IgG, IgM MPO ELISA

Serum from patients and healthy participants were incubated on QUANTA Lite MPO SC Kits (INOVA) at a 1:100 dilution for 30 minutes at room temperature for the IgG assays following manufacturer protocols. For the IgM ELISA, serum from patients and healthy participants were incubated on QUANTA Lite MPO SC Kits (INOVA) at a 1:100 dilution for 2 hours. ELISAs were then incubated with an anti-human IgM conjugated with alkaline phosphatase (μ-chain specific–alkaline) secondary antibody at a 1:2,000 dilution for 1 hour at room temperature and then washed using the INOVA wash buffer. Following the washing step, 1-Step PNPP solution (Cat 37621, Thermo Fisher Scientific) was used to detect the IgM secondary antibody. Plate-to-plate variation was controlled by calculating each patient as a percentage of a positive control, which was run on each plate. All samples were run in duplicate, and all duplicates were within 20% of each other.

### IgM and IgG binding assay

Native MPO or hydralazine-modified MPO (2.5 μg/mL) were coated to a 96-well microtiter plate (Cat 3855, Thermo Fisher Scientific) with coating buffer (100 mM bicarbonate/carbonate) and incubated at the cool room overnight. Plates were washed 3 times with washing buffer (PBS with 0.05% Tween-20) before blocking with the blocking buffer (1% BSA in PBS). Purified IgM or IgG (1.0 μg/mL) from individual patients was applied with blocking buffer and incubated at 4°C overnight. 3 additional washes were performed with washing buffer before applying anti-human IgG or IgM secondary antibody conjugated with horseradish peroxidase. After 1 hour incubation at room temperature, 100 μL of 1-step slow TMB-ELISA substrate solution (Cat 34024, Thermo Fisher Scientific) was applied to each well. After sufficient color development, 100 μL stop solution (0.2 M H_2_SO_4_) was applied and absorbance was recorded in a microplate reader at 450 nm.

### Neutrophil-like HL-60 oxidative burst assay

HL-60 cells (ATCC, CCL240) were cultured in complete RPMI, which contained Glutamax, penicillin- streptomycin, and 10% FBS at 37°C in 5% CO_2_ and passaged at least every 3 days to maintain cells at 2 × 10^5^ cells/mL. The cells were differentiated to become “neutrophil-like” by plating cells at 2 × 10^5^ cells/mL in complete RPMI supplemented with 1.3% Hybri-max DMSO (Sigma-Aldrich), and 100 ng/mL carrier-free Granulocyte Colony Stimulating Factor (Biolegend). Cells were incubated for 6 days at 37°C in 5% CO_2_ undisturbed (45). 1 × 10^5^ differentiated HL-60 cells were seeded each well of a 96 wells plate (glass bottom black plate, Cat 655892, Greniner Bio-one) with 200 μL HBSS buffer. The cells were primed with cytochalasin B (2 μM) for 5 minutes before adding PMA (100 ng/mL) or IgM (0.75 μg/well) isolated from individuals in the healthy control group, patients with nonhydralazine associated ANCA, or patients with hydralazine-associated ANCA. ROS generation was detected by DHE (2 μM) and plates were read by TECAN with EX 510 nm/EM 610 nm.

### Animal studies

#### Mice.

C57BL/6 (B6) MPO^–/–^ mice were originally generated by Aratani et al. (46). B6 and Rag2^–/–^ with B6 background (RAGN12) mice were purchased from Taconic Biosciences. Mice were maintained by the animal facility of the University of North Carolina.

### Immunization and adoptive splenocytes transfer

Immunization and splenocyte transfer were performed as previously reported (41). MPO^–/–^ and WT B6 mice were immunized intraperitoneally with 20 μg rmMPO or HA-rmMPO in CFA and boosted twice by intraperitoneal injection of 10 μg of corresponding antigen in IFA. Four days after the second boost, splenocytes were isolated from immunized MPO^–/–^ and WT mice by homogenizing the spleens in cold RPMI 1640 medium and then washing twice with RPMI 1640. Red blood cells were removed with lysis buffer (Sigma-Aldrich) followed by washing with RPMI 1640 and then suspended in sterile RPMI 1640. Suspensions of 5 × 10^7^ splenocytes were administered intravenously to Rag2^–/–^ mice. Development of circulating antibodies was monitored on days 0, 4, 7, 11, and 14 after anti-MPO splenocyte transfer by anti-MPO ELISA (41). On day 14 after splenocyte transfer, mice were sacrificed and kidney tissues were collected and examined by light microscopy. Crescents and necrosis were expressed as mean percentage of glomeruli with lesions (41).

### ELISpot assays

ELISpots for mouse IFN-γ and IL-17A were purchased from MabTech. Plates were washed with sterile PBS and blocked with RPMI + 10% FBS for a minimum of 30 minutes. Total splenocytes (*n* = 3 mouse spleens combined per treatment) were plated at 300,000 cells per well. Stimulating conditions were added simultaneously to include: Mock (cells + media only), cell stimulation cocktail (Biolegend), and then either native MPO or hydralazine-modified MPO at 3, 6, or 12 μg/mL. Plates were incubated at 37°C for 18 hours. After overnight incubation, cells were dumped and plates were washed extensively. Manufacturer’s protocols were followed for detection antibody, streptavidin-alkaline phosphatase, and substrate solution. Color development was stopped by extensive washing under tap water. Plates were allowed to dry at ambient temperature for 24 hours before imaging and counting in an ELISpot reader.

### Statistics

Categorical measures were expressed as both numerical values and percentages, while continuous measures were represented as mean and SD)or median and IQR, in cases of nonnormal distribution. Fisher’s exact test was employed for comparing categorical variables; Wilcoxon’s 2-Sample Test or Kruskal-Wallis Test was used for continuous variables. The comparison of patients’ plasma anti-MPO IgM and IgG values were compared using Mann-Whitney test. Densitometry analysis of Western blot results was conducted using NIH Image J (Version 1.53K). To compare variables between 2 groups, the Mann-Whitney test was utilized, and for multiple group comparisons, Dunn’s multiple comparisons test was applied. Animal study data were expressed as the mean ± SEM and statistically analyzed using 1-way ANOVA for multiple comparisons. The multiple comparisons of IgM/IgG for any 2 groups of hydralazine associated, nonhydralazine-associated patients and healthy participants were calculated by Dunn’s multiple comparisons test. Statistical significance was defined as *P* < 0.05. All statistical analyses were performed, and figures were generated using SAS (Version 9.4, SAS Institute) and GraphPad Prism (Version 9.5.1 for Windows, GraphPad Software).

### Study approval

Our study protocols adhered to and received approval from the University of North Carolina Institutional Review Board (IRB no. 21-1913) (Chapel Hill, North Carolina, USA), and all patients provided written, informed consent prior to participation.

All animal procedures were approved by the University of North Carolina at Chapel Hill IACUC. Animal experiments were conducted in accordance with the NIH Guide for the Care and Use of Laboratory Animals, 8th ed, National Academies Press, 2011.

### Data availability

Raw data are provided in the supplemental Supporting Data Values file. The authors declare that all data supporting the findings of this study are available will be available from the corresponding authors upon request. Human participant data will be available deidentified.

## Author contributions

RJF and GX conceptualized the project. RJF, MEF, and JCJ acquired funding for the project. GX, PH, EAM, OL, and JSP developed the methodology and performed the experiments. DPC, EMZ, EYW, NMO, VKD and RJF performed patients’ chart reviews. PH and JCJ performed histopathologic evaluation of kidney lesions. GX, EAM and OL drafted the manuscript. All authors edited the manuscript.

## Supplementary Material

Supplemental data

Unedited blot and gel images

Supporting data values

## Figures and Tables

**Table 1:**
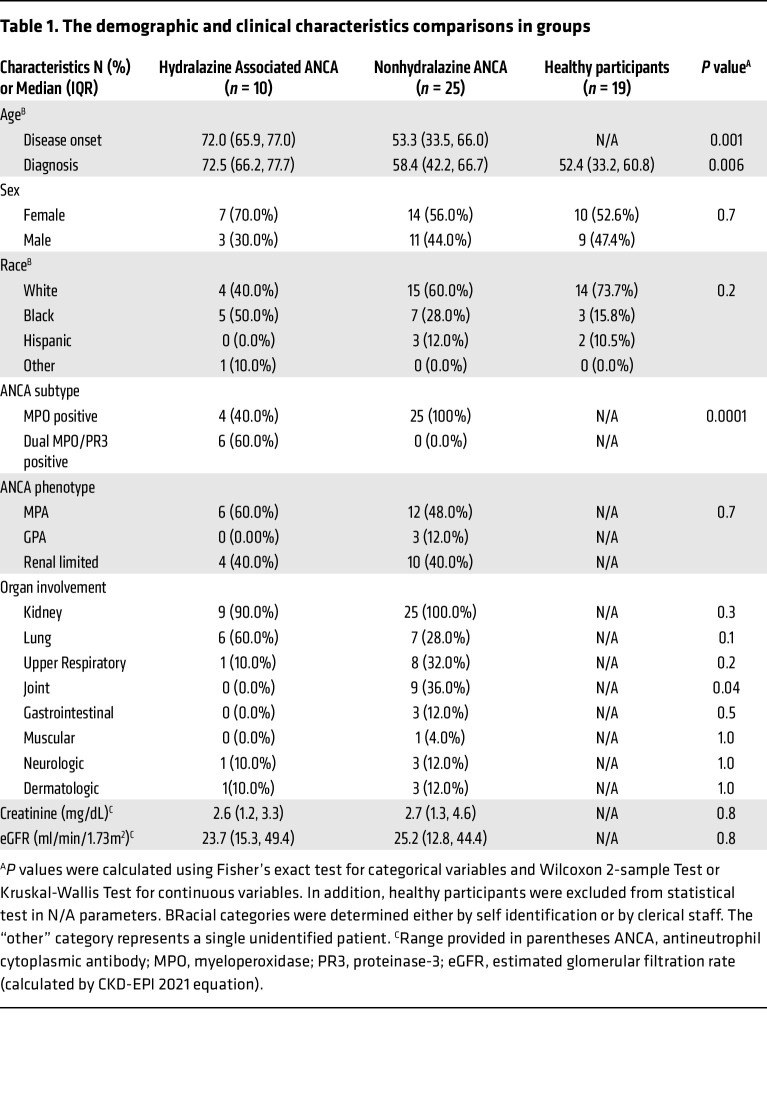
The demographic and clinical characteristics comparisons in groups

## Version 1 02/28/2025

In-Press Preview

## Version 2 04/15/2025

Electronic publication
