# Supplementary material for: Sequential carbonyl derivatives and hydrazone adduct formation on myeloperoxidase contribute to development of ANCA vasculitis
Source: J Clin Invest. 2025 Feb 27;135(8):e178813. doi: 10.1172/JCI178813 (PMC11996859; doi:10.1172/JCI178813)
Supplement: Supplemental data [file jci-135-178813-s041.pdf]

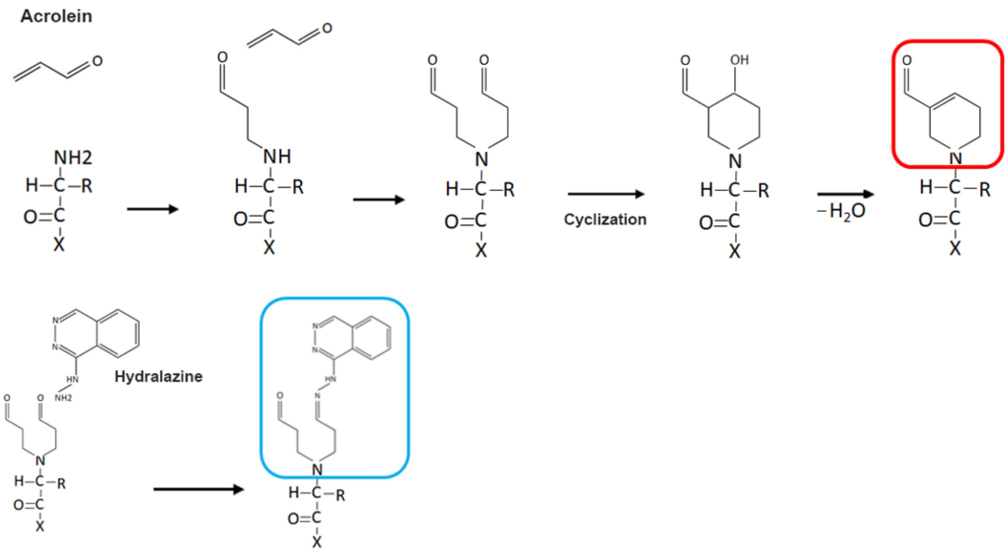

Supplemental Figure 1: Carbonyl derivatives and hydrazone adduct formation in the presence of acrolein. Acrolein is a highly reactive unsaturated aldehyde, which reacts preferentially with amine group containing amino acids, such as lysine and histidine residues, via Michael-type addition reactions, preserving the aldehyde functionality on the modified protein. The major adducts formed on reaction with protein is *N*$^{\varepsilon}$-3-formyl-3,4-dehydropiperidino lysine (FDP-lysine) (red square). Michael-type addition products can covalently bind to hydralazine through the retained electrophilic carbonyl moiety. One prominent product formed upon the addition of hydralazine to acrolein-modified protein is a bis-ACR-lysine-hydrazone adduct (blue square).

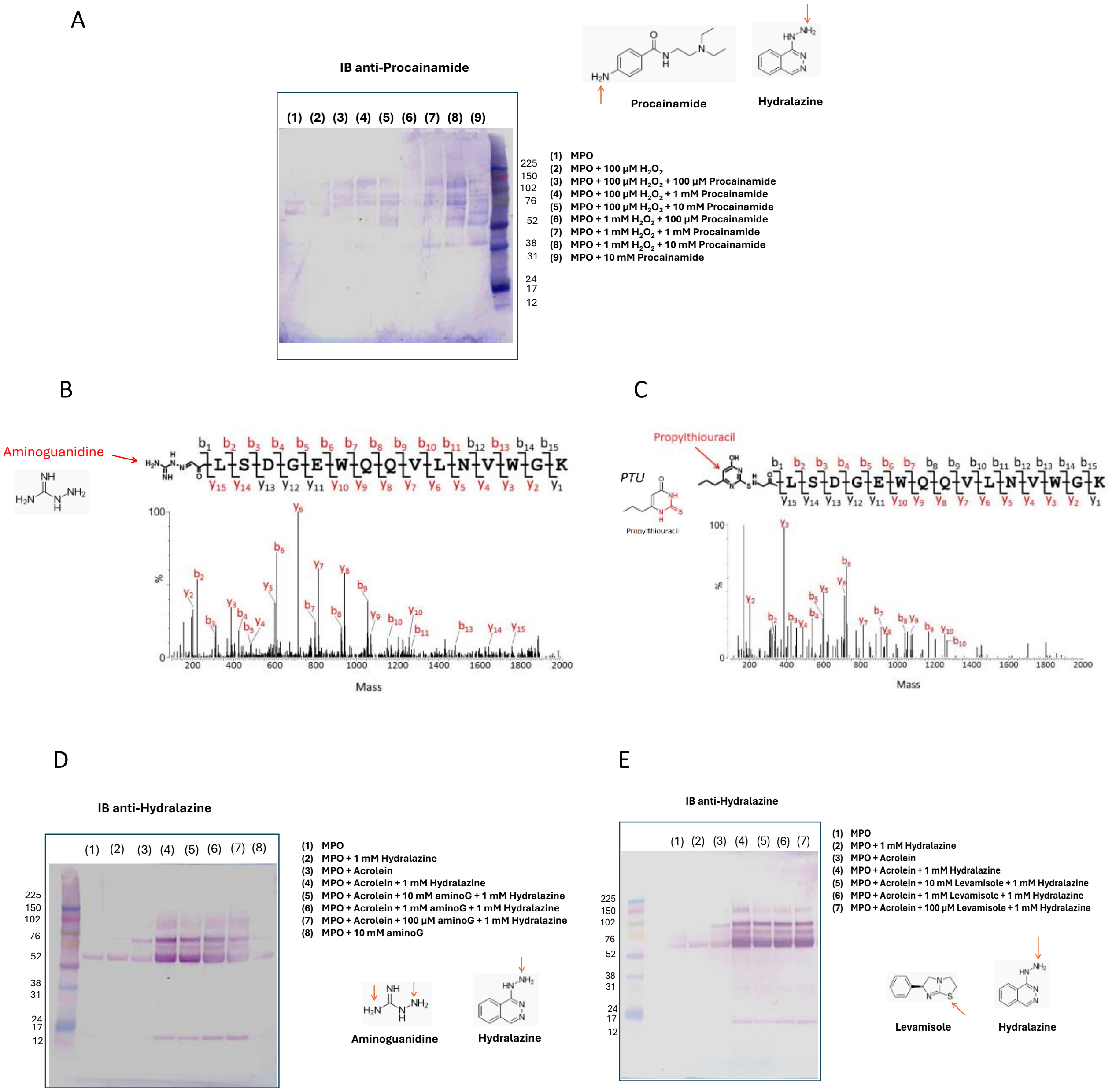

Supplemental Figure 2: Tests of different drugs binding to myeloperoxidase (MPO) or myoglobin. (A) Different incubation conditions of procainamide with MPO affects the ability of adduct formation on MPO. (B) ESI-QTOF mass spectrum of an aminoguanidine-myoglobin adduct. The N-terminal amino acid Gly-1 was identified as the main site of Drug-Protein adduction. (C) ESI-QTOF mass spectrum of propylthiouracil-myoglobin adduct. The N-terminal amino acid Gly-1 was identified as the main site involved in the formation of Propylthiouracil-protein adduct. (D) Aminoguanidine, which binds to MPO via the same mechanism as hydralazine, was able to competitively attenuate hydralazine associated with MPO in a reverse dose dependent manner (lane 7 vs lane 4, 5 and 6). (E) Levamisole, which binds to MPO via a different mechanism from hydralazine, was unable to attenuate hydralazine associated with MPO in all tested concentrations (lane 4, 5, 6 and 7).

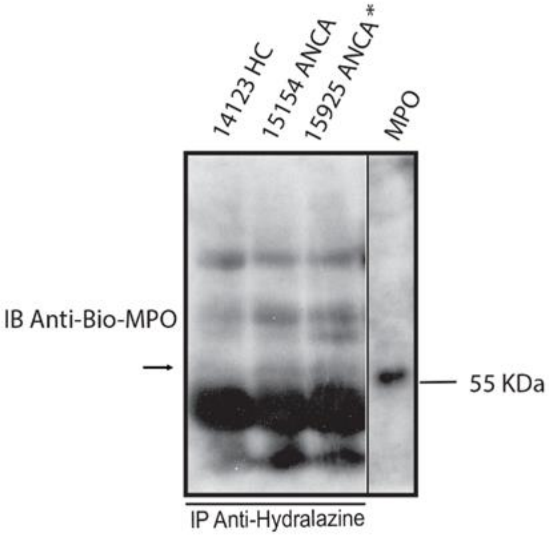

Supplemental Figure 3: Hydrazone adduct could not be detected in circulating myeloperoxidase (MPO) from non-hydralazine-associated patients. The patient noted with asterisk was initially accidently grouped as a hydralazine-associated patient; therefore, this sample was included with one healthy subject (HC) and one non-hydralazine-associated patient (ANCA) for a pulldown assay. No myeloperoxidase signal was detected in pulldown of hydralazine-modified proteins in any of these samples. The lanes were run on the same gel but were noncontiguous. Arrow indicates the expected position of MPO.

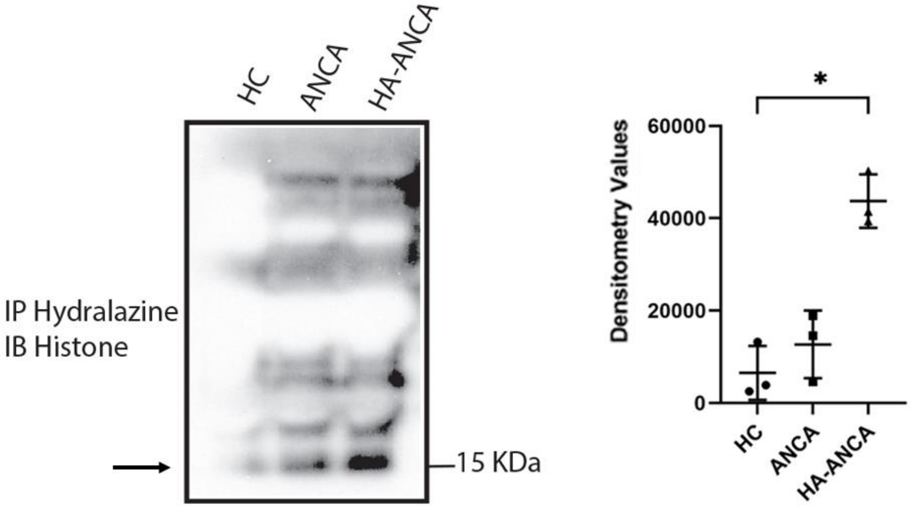

Supplemental Figure 4: Hydrazone adduct was detected on histones from hydralazine-associated patients. Plasma from healthy subject (HC), non-hydralazine-associated ANCA (ANCA) and hydralazine-associated ANCA (HA-ANCA) patients were immunoprecipitated with anti-hydralazine antibody and immunoblotted with anti-histone antibody (IBL America, Cat#2112442) (n=3). Densitometry values were obtained using Image J (NIH, 1.53K version) and graph was drawn using GraphPad Prism (GraphPad Software, Version 9.5.1). *P<0.05 assessed by Dunn's multiple comparisons test.

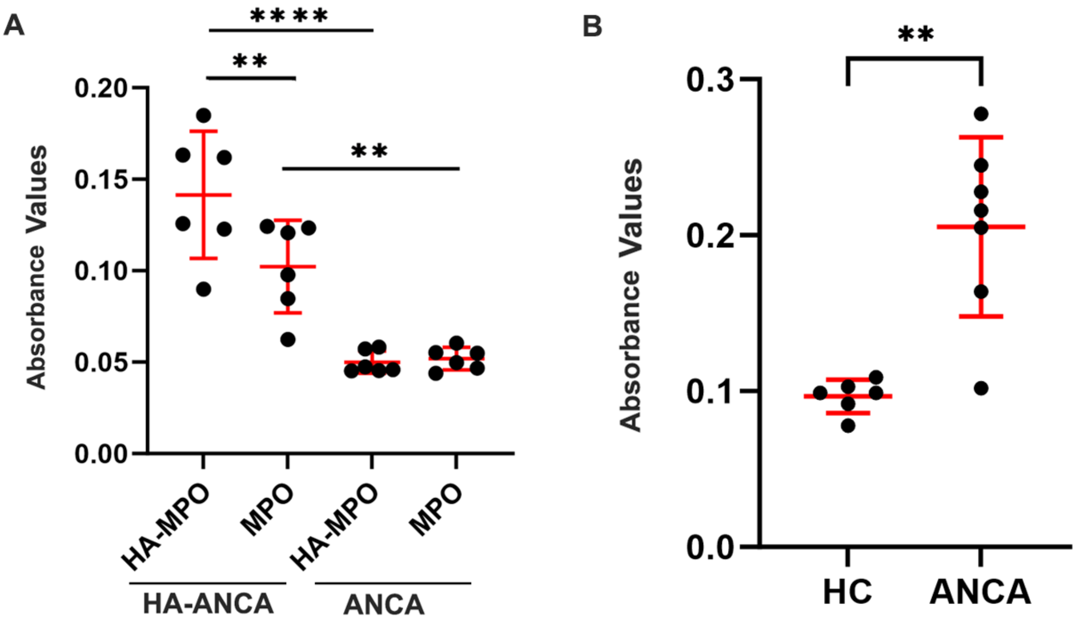

Supplemental Figure 5: Different affinity to hydralazine-modified myeloperoxidase (HA-MPO) or native MPO of immunoglobins purified from hydralazine-associated patients (HA-ANCA) or non-hydralazine-associated patients (ANCA) or health controls (HC). HA-MPO or native MPO (250 ng/well) (A) or native MPO only (250 ng/well) (B) were coated on the ELISA plates with coating buffer for overnight incubation in the cool room. IgM (A) or IgG (B) (100ng/well) purified from HA-ANCA (n=6) or non-hydralazine associated ANCA (n=6 for panel A; n=7 for panel B) patients or HC (n=6) following the protocol in the method section was applied to test their binding ability. HRP-conjugated anti-human IgM (0.2 ug/ml) (A) or IgG (0.13 ug/ml) (B) was applied for 1hr incubation at room temperature and analyzed spectrophotometrically at OD 450 nm. Graphs were drawn using GraphPad Prism (GraphPad Software, Version 9.5.1). **P<0.01; ****P<0.0001 assessed by one way ANOVA multiple comparisons test (A) or Mann-Whitney test (B).

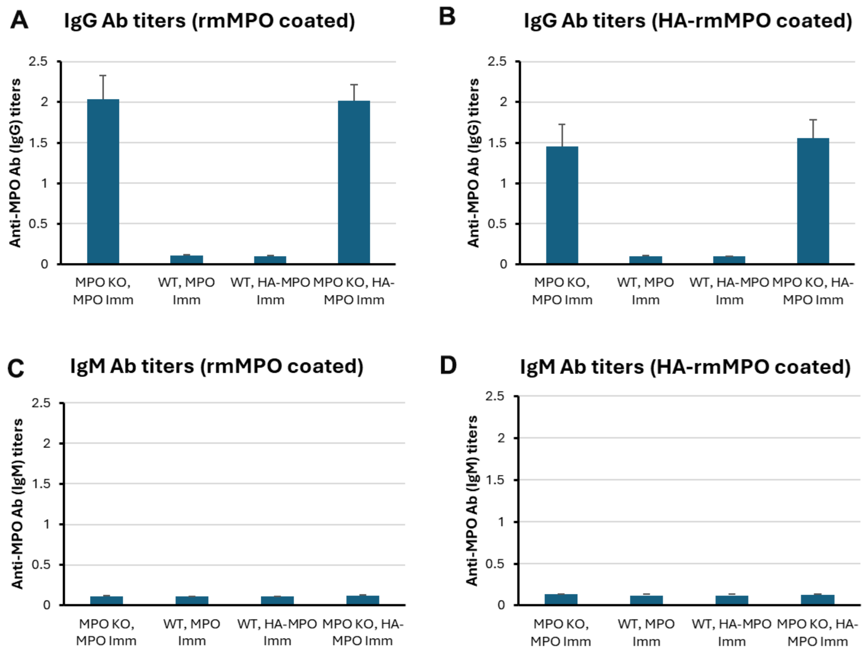

Supplemental Figure 6: Antibodies generated in mice immunized with recombinant mouse MPO (rmMPO) or hydralazine-modified rmMPO (HA-rmMPO). Circulating anti-rmMPO or anti-HA-rmMPO antibodies were measured using indirect enzyme linked immunosorbent assay (ELISA). For the ELISA, microtiter plates were coated with 0.25 µg per well with rmMPO (A and C) or HA-rmMPO (B and D), incubated with 2000-fold dilutions of mouse sera, developed with AP–conjugated goat antibodies specific for mouse IgG (A and B) or mouse IgM (C and D), and analyzed spectrophotometrically at OD 405 nm.

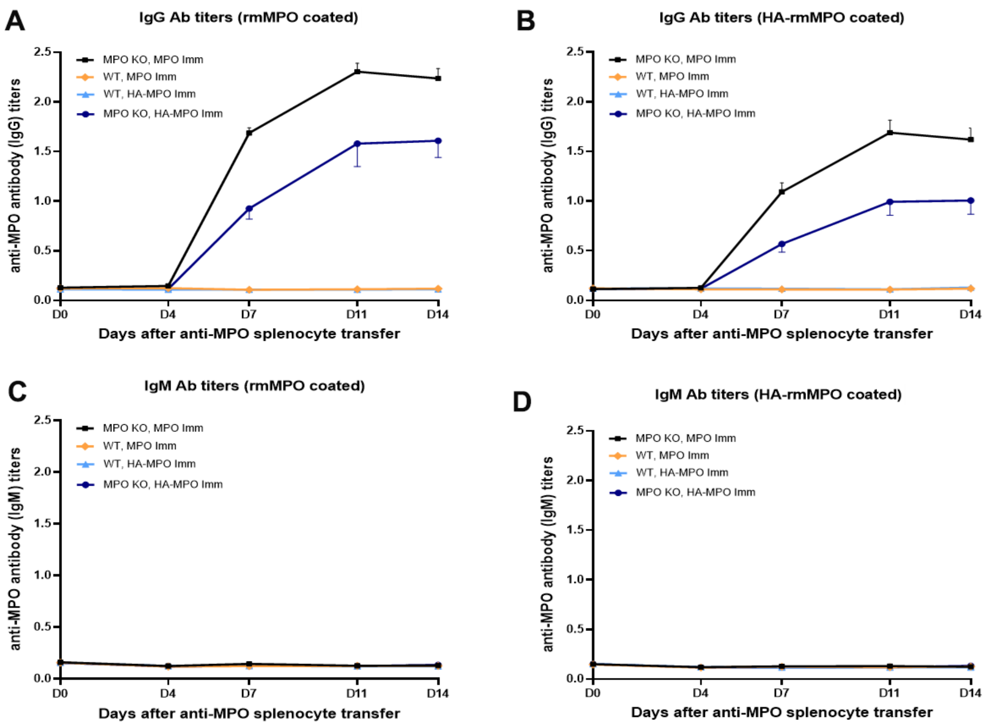

Supplemental Figure 7: Antibodies generated in rag2-/- mice after splenocyte transfer from recombinant mouse MPO (rmMPO) or hydralazine-modified rmMPO (HA-rmMPO) immunized mice. Circulating anti-rmMPO or anti-HA-rmMPO were measured by indirect enzyme linked immunosorbent assay (ELISA). For the ELISA, microtiter plates were coated with 0.25 µg per well rmMPO (A and C) or HA-rmMPO (B and D), incubated with 2000-fold dilutions of mouse sera from rag2-/- mice after splenocyte transfer, developed with AP–conjugated goat antibodies specific for mouse IgG (A and B) or mouse IgM (C and D), and analyzed spectrophotometrically at OD 405 nm.

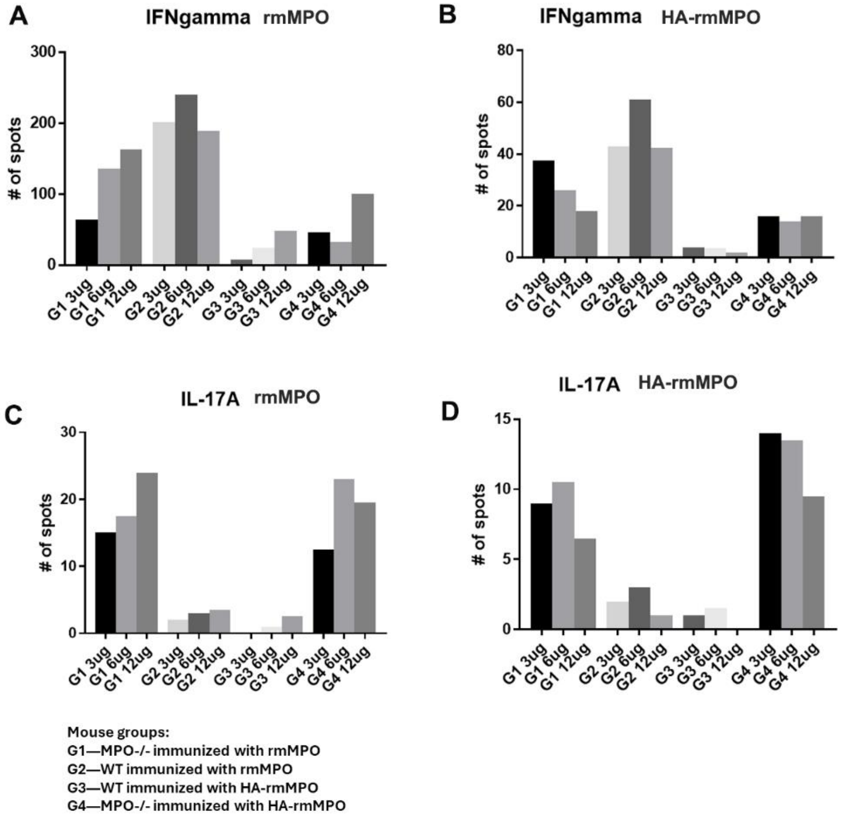

Supplemental Figure 8: T cell production of IFN gamma and IL-17A in response to native recombinant mouse MPO (rmMPO) and hydralazine-modified rmMPO (HA-rmMPO). ELISpots for mouse IFN gamma (A and B) and IL-17A (C and D) were utilized to determine T cell responses from mice immunized with either native rmMPO or HA-rmMPO. IFN gamma was produced by T cells in response to either rmMPO or HA-rmMPO in Groups 1, 2, and 4, indicative of recall response. IL-17A production from T cells in response to rmMPO or HA-rmMPO was limited to MPO-/- mice that had been previously immunized.

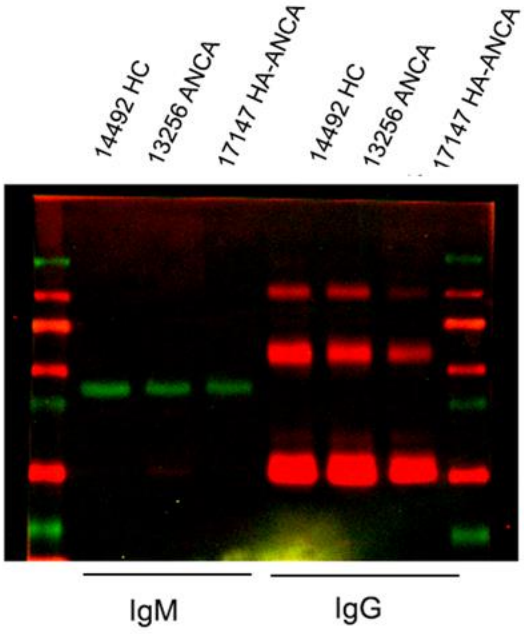

Supplemental Figure 9: Purity of isolated IgM and IgG. IgM and IgG were isolated following the protocol described in Methods. The isolated IgG and IgM were loaded and separated using a 9% SDS PAGE gel and transferred to a nitrocellulose membrane. The membrane was incubated with a mixture of anti-human IgG (conjugated with IRDye®680) and anti-human IgM (conjugated with IRDye®800) secondary antibodies.
