# Supplementary material for: Sequential carbonyl derivatives and hydrazone adduct formation on myeloperoxidase contribute to development of ANCA vasculitis
Source: J Clin Invest. 2025 Feb 27;135(8):e178813. doi: 10.1172/JCI178813 (PMC11996859; doi:10.1172/JCI178813)
Supplement: Unedited blot and gel images [file jci-135-178813-s042.pdf]

Figure 1D

IB DNP

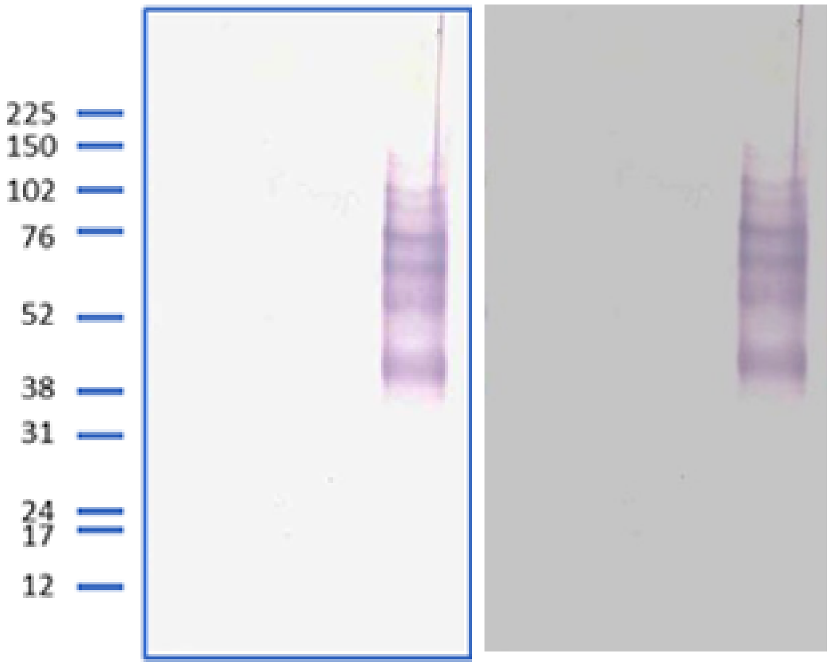

Figure 1E

IB Hydralazine

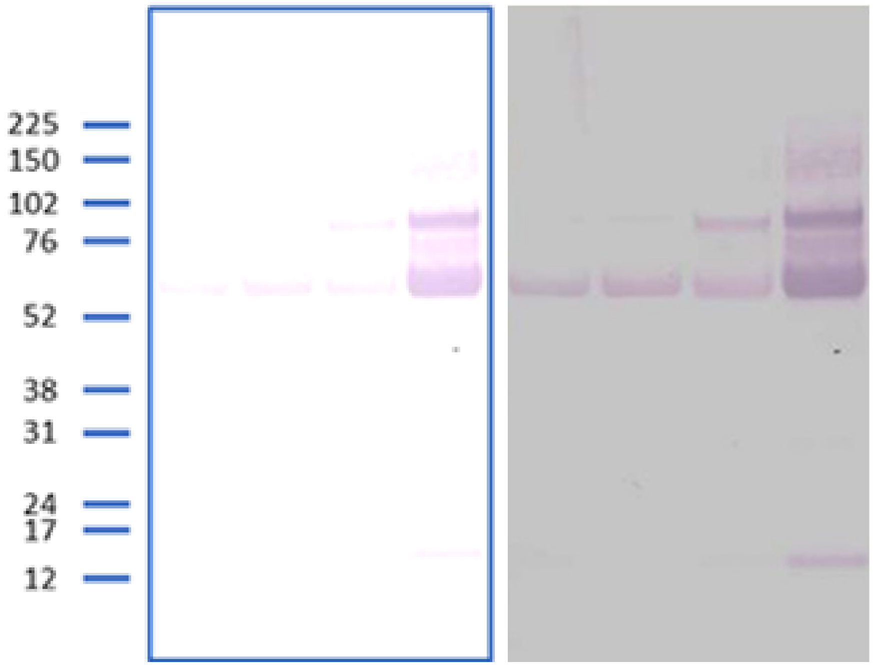

Full unedited gels for Figure 1D and E

Figure 2A gel 1

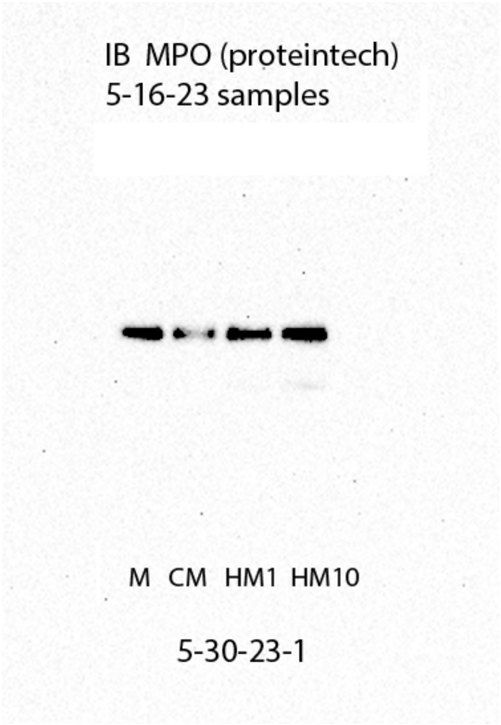

Figure 2A gel 2

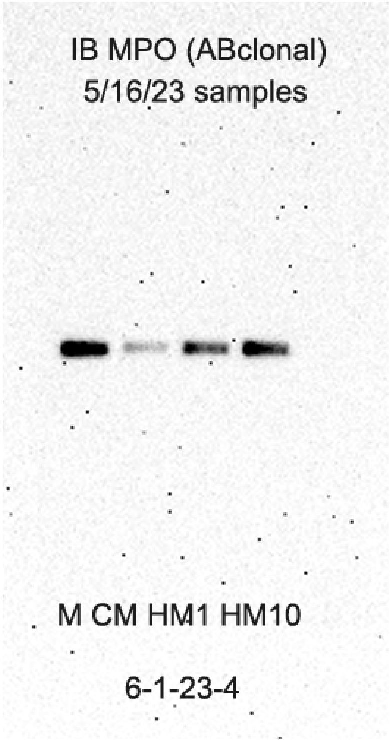

Figure 2A gel 3

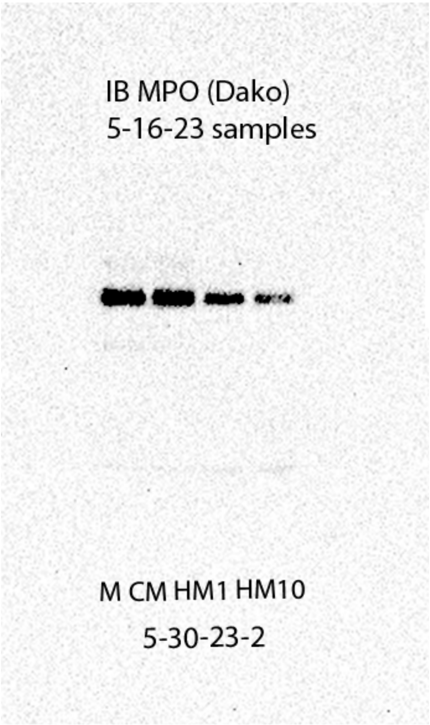

Figure 2A gel 4

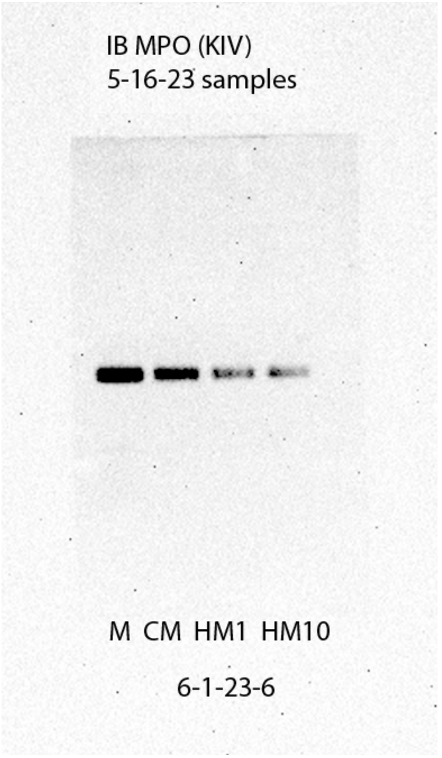

Figure 2A gel 5

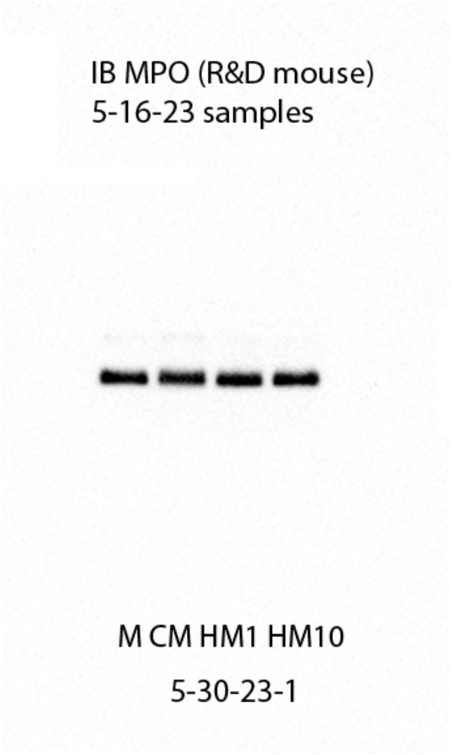

Full unedited gels for Figure 2A

Figure 2 Gel 6

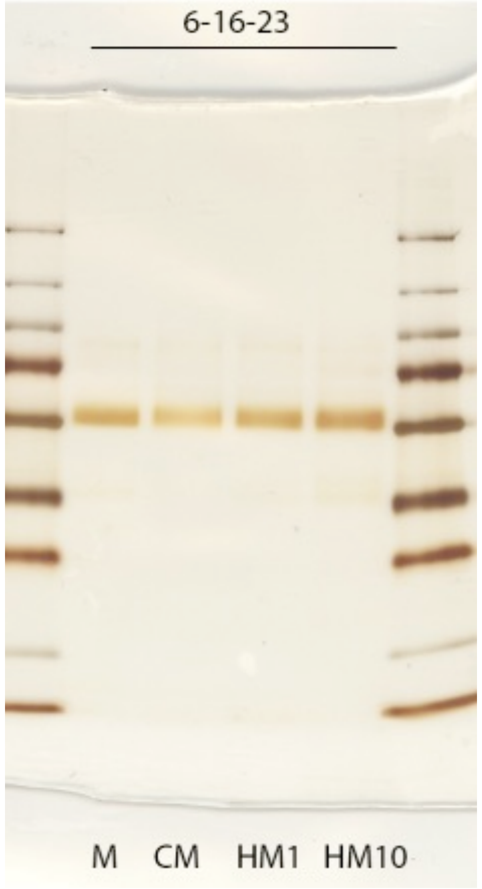

Full unedited gels for Figure 2A gel 6

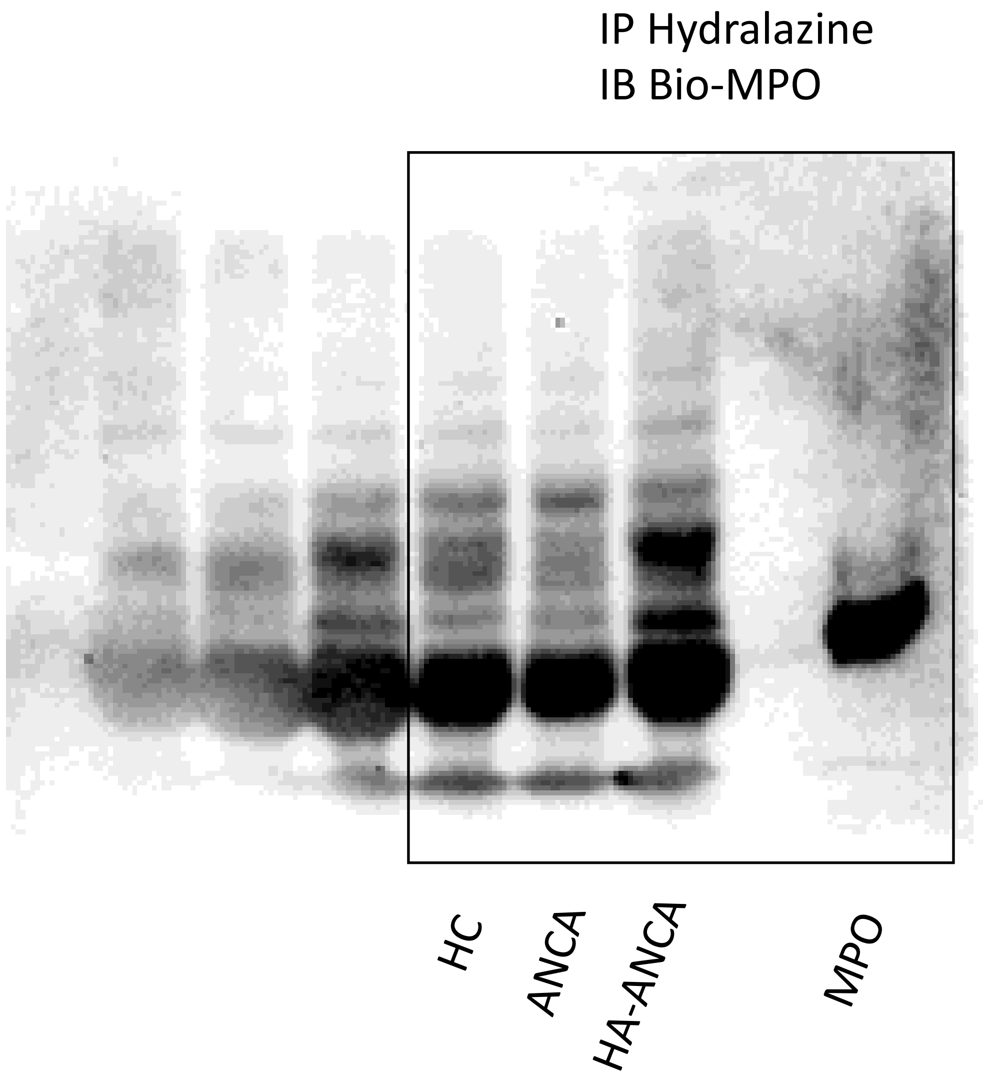

Full unedited gels for Figure 3A

HC ANCA HA-ANCA IB Purified patient's IgM

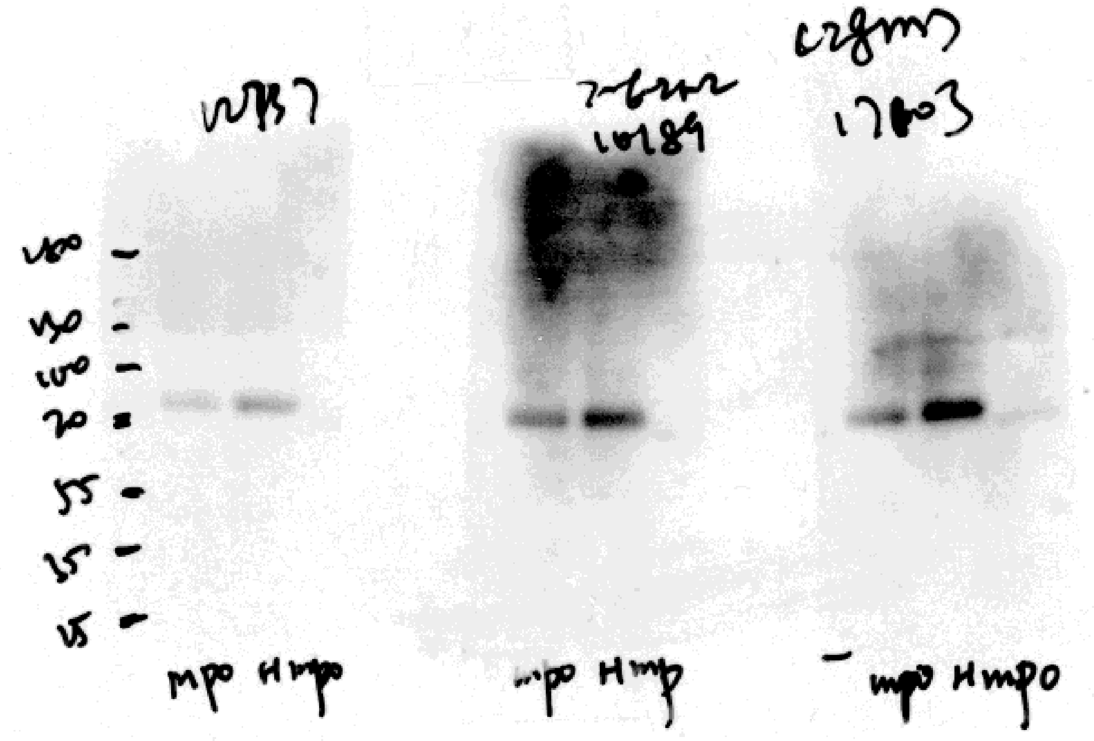

MPO HMPO MPO HMPO MPO HMPO

Full unedited gels for Figure 4B

HC ANCA HA-ANCA IB Purified patient's IgG

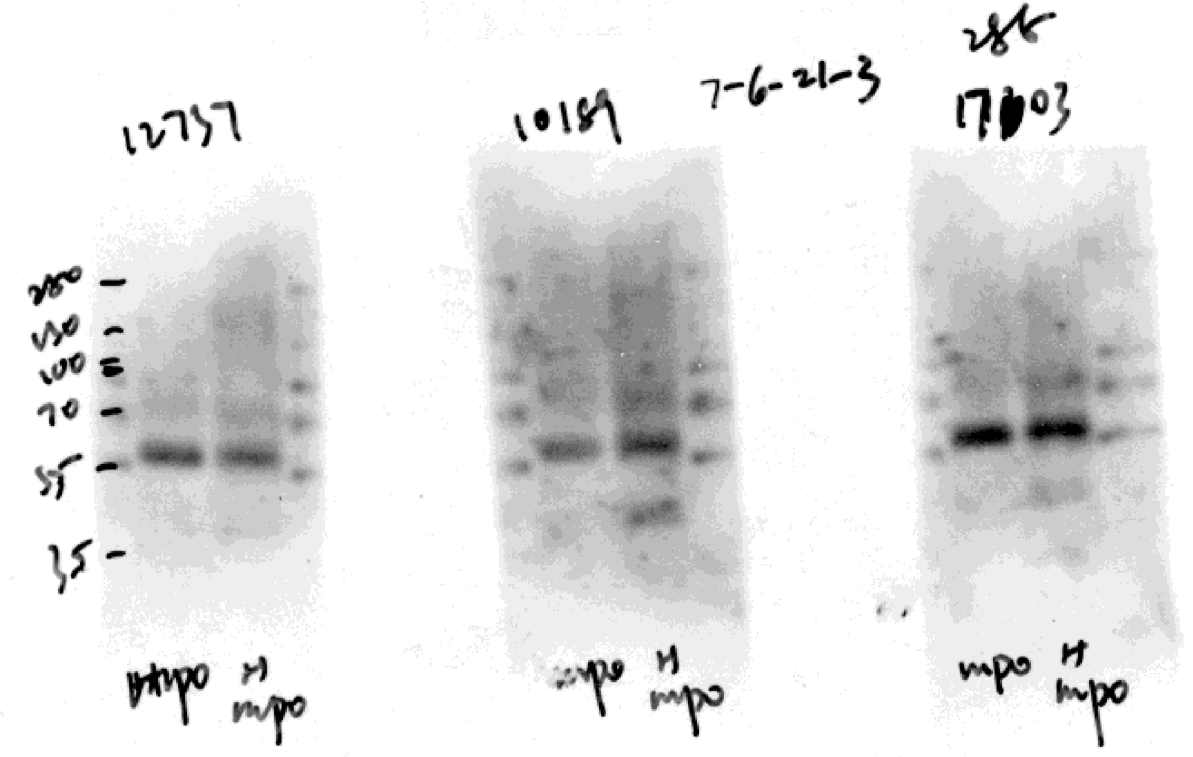

MPO HMPO MPO HMPO MPO HMPO

Full unedited gels for Figure 4C

IB anti-Procainamide

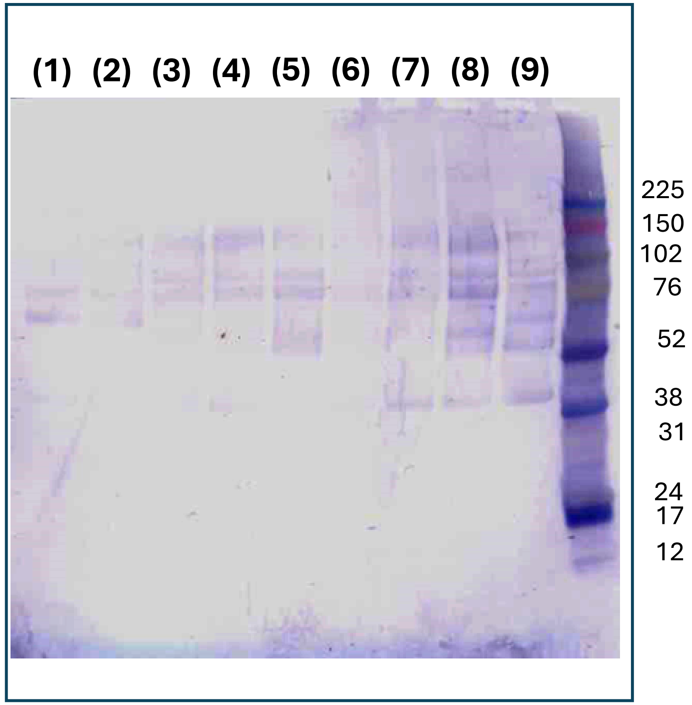

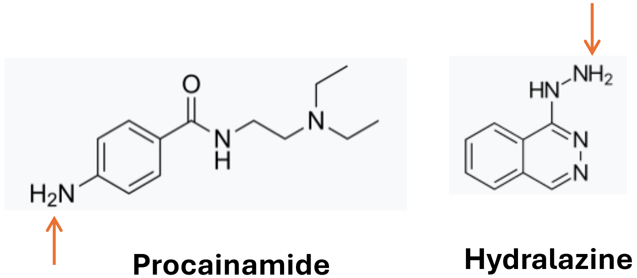

Procainamide **Hydralazine**

(1) MPO
(2) MPO + 100 µM H_2O_2
(3) MPO + 100 µM H_2O_2 + 100 µM Procainamide
(4) MPO + 100 µM H_2O_2 + 1 mM Procainamide
(5) MPO + 100 µM H_2O_2 + 10 mM Procainamide
(6) MPO + 1 mM H_2O_2 + 100 µM Procainamide
(7) MPO + 1 mM H_2O_2 + 1 mM Procainamide
(8) MPO + 1 mM H_2O_2 + 10 mM Procainamide
(9) MPO + 10 mM Procainamide

Full unedited gels for Supplemental Figure 2A

IB anti-Hydralazine

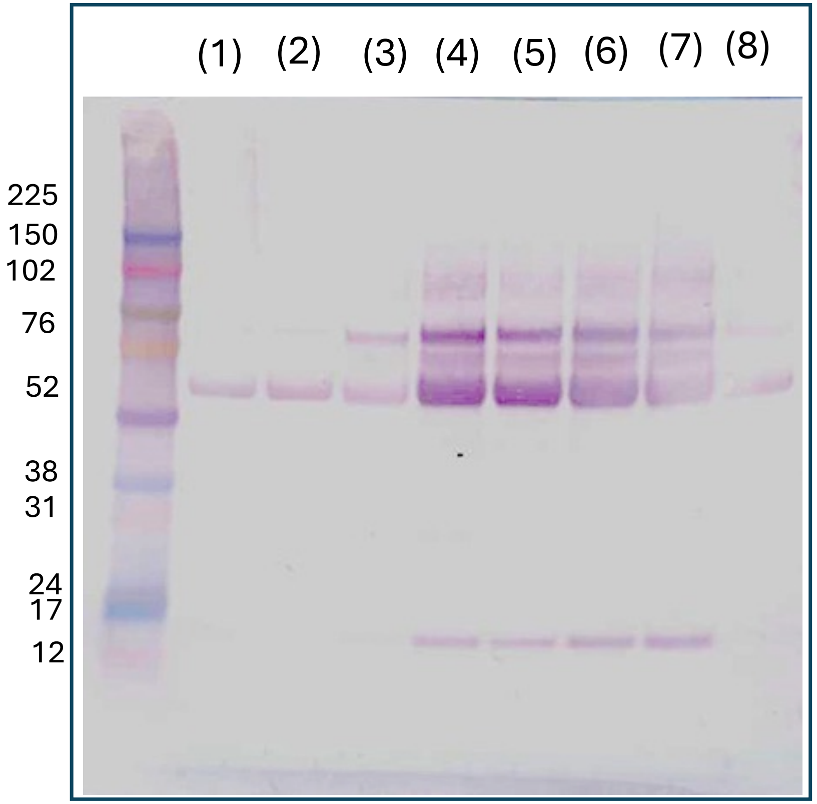

(1) **MPO**
(2) **MPO + 1 mM Hydralazine**
(3) **MPO + Acrolein**
(4) **MPO + Acrolein + 1 mM Hydralazine**
(5) **MPO + Acrolein + 10 mM aminoG + 1 mM Hydralazine**
(6) **MPO + Acrolein + 1 mM aminoG + 1 mM Hydralazine**
(7) **MPO + Acrolein + 100 μM aminoG + 1 mM Hydralazine**
(8) **MPO + 10 mM aminoG**

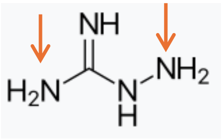

Aminoguanidine

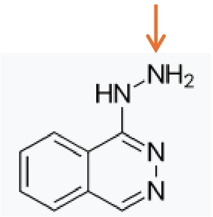

Hydralazine

Full unedited gels for Supplemental Figure 2D

IB anti-Hydralazine

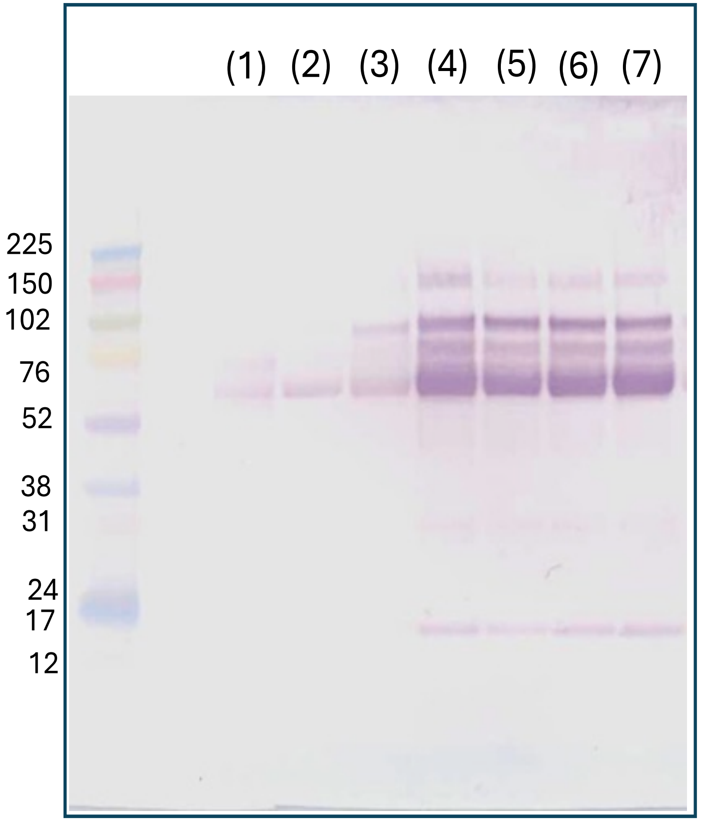

(1) MPO
(2) MPO + 1 mM Hydralazine
(3) MPO + Acrolein
(4) MPO + Acrolein + 1 mM Hydralazine
(5) MPO + Acrolein + 10 mM Levamisole + 1 mM Hydralazine
(6) MPO + Acrolein + 1 mM Levamisole + 1 mM Hydralazine
(7) MPO + Acrolein + 100 μM Levamisole + 1 mM Hydralazine

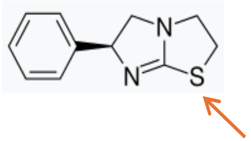

Levamisole

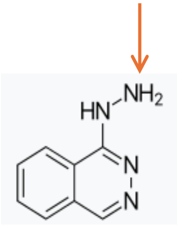

Hydralazine

Full unedited gels for Supplemental Figure 2E

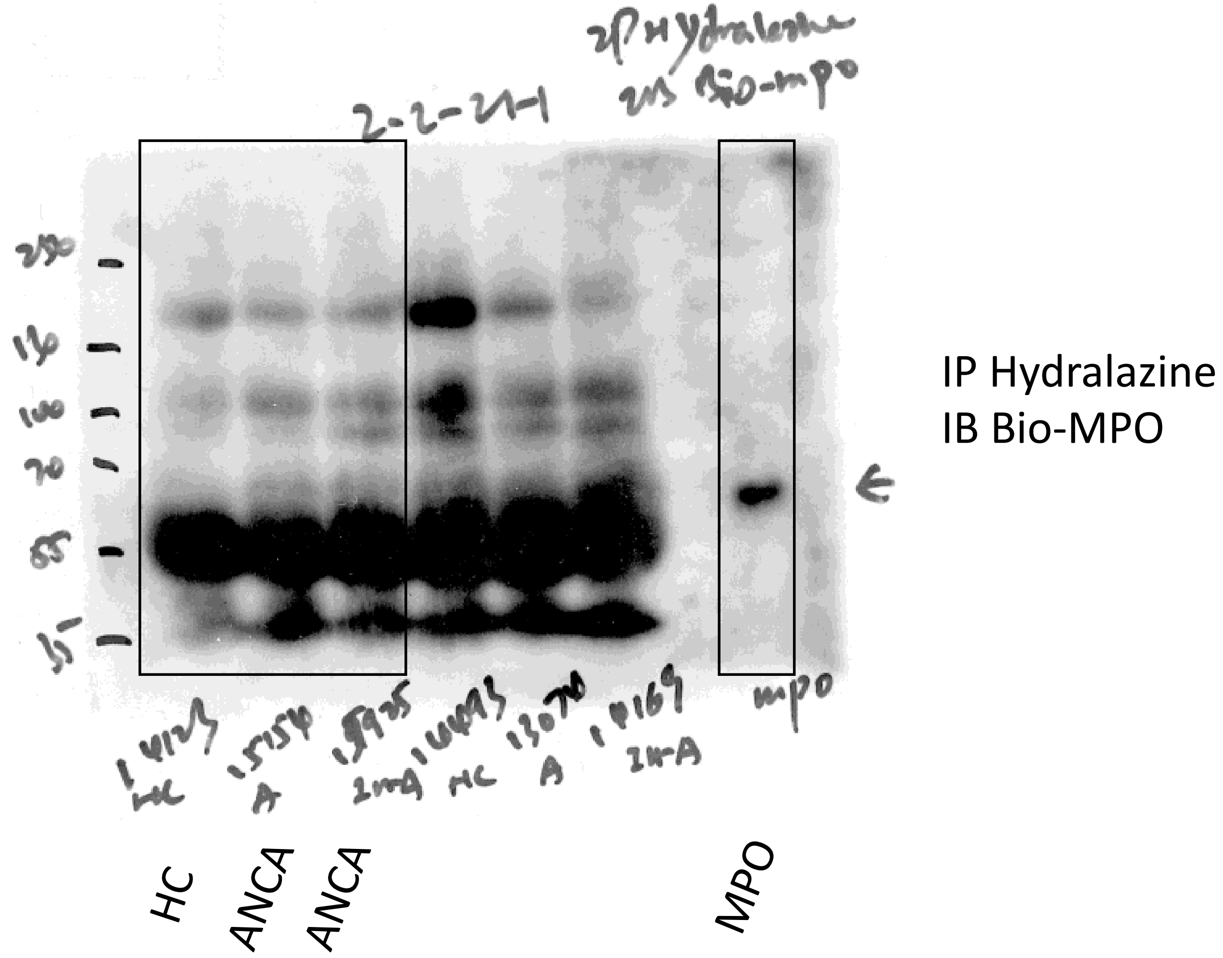

Full unedited gels for Supplemental Figure 3

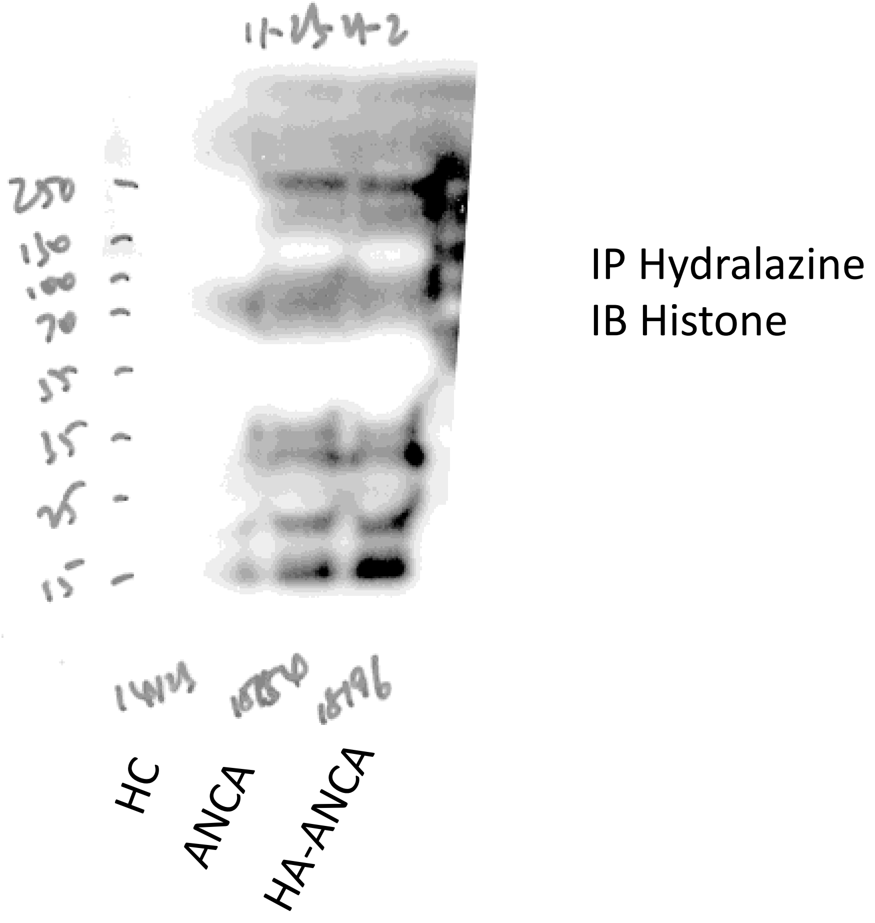

Full unedited gels for Supplemental Figure 4A

IB a mixture of anti-human IgM conjugated with IRDye®800 and anti-human IgG conjugated with IRDye®680

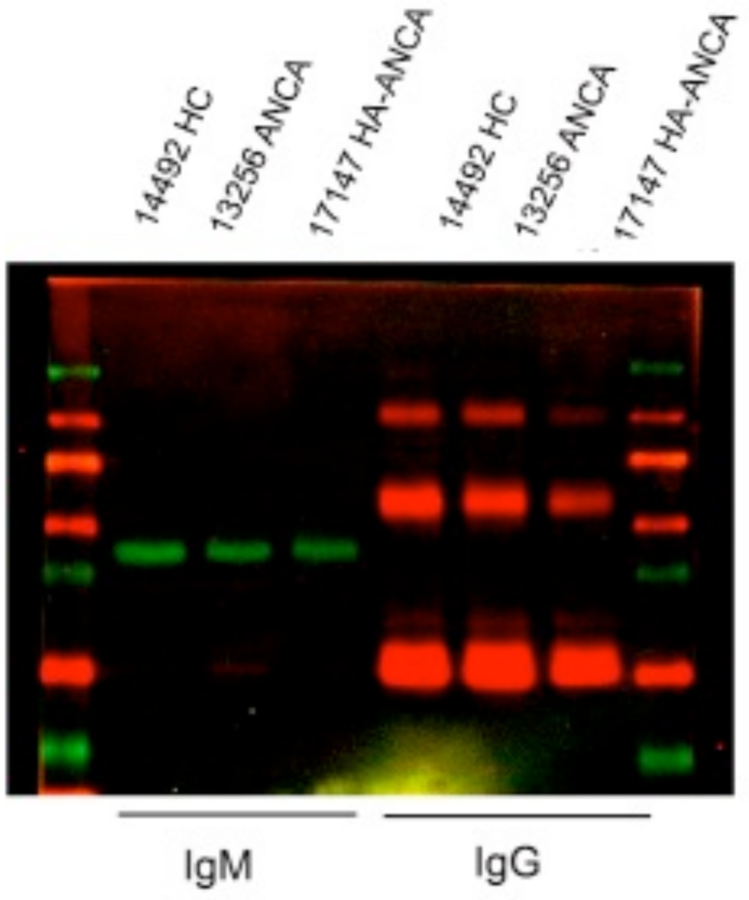

Full unedited gels for Supplemental Figure 9
